# Investigating the causal relationship between 731 immune phenotypes and thyroid cancer risk: A bidirectional Mendelian randomization study

**DOI:** 10.1097/MD.0000000000045072

**Published:** 2025-10-17

**Authors:** Tingting Bian, Yali Zhang, Weiyi Lai, Jianguo Zhang, Daishan Jiang, Yifei Liu

**Affiliations:** a Department of Pathology, Affiliated Hospital of Nantong University, Nantong, China; b School of Medicine, Nantong University, Nantong, China; c Department of Emergency Medicine, Affiliated Hospital of Nantong University, Nantong, China.

**Keywords:** causal inference, immunocyte phenotype, Mendelian randomization, thyroid cancer

## Abstract

Previous studies have demonstrated a significant correlation between immune cells and thyroid cancer (TC). Nevertheless, there remains uncertainty regarding whether this association indicates a causal relationship. We performed a bidirectional Mendelian randomization (MR) analysis to explore the causal relationship between 731 immune phenotypes and thyroid cancer. Our primary analytical method was the inverse variance weighting technique, complemented by supporting analyses using weighted median, MR-Egger, simple mode, and weighted mode. Our results were also robustly assessed using sensitivity analyses to account for heterogeneity and potential horizontal pleiotropy. The results from the inverse variance weighting analysis, which examined 7 groups of immune cells in their antithyroid cancer effects, indicated that 11 immune cell traits were positively correlated with the occurrence and progression of thyroid cancer (odds ratio [OR] > 1, *P* < .05), while 22 immune cell traits showed a negative correlation (OR < 1, *P* < .05). In the reverse MR analysis, thyroid cancer was positively associated with 2 immune cell phenotypes (*P* < .05, OR > 1) and negatively associated with 1 immune cell phenotype (*P* < .05, OR < 1). None of these findings displayed evidence of heterogeneity, horizontal pleiotropy, or reverse causality (*P* > .05). This research offers a perspective on the biological mechanisms between thyroid cancer and immune cells, contributing to the exploration of early intervention and treatment options.

## 1. Introduction

Over the past few decades, the worldwide occurrence of thyroid cancer has been increasing steadily. This upward trend has led to thyroid cancer becoming the most frequently diagnosed endocrine malignancy, surpassing other endocrine-related cancers in both prevalence and impact on public health.^[1]^ Thyroid cancer is treated primarily by surgery, radioactive iodine therapy, and hormone replacement therapy.^[2]^ While early detection and proper treatment often result in favorable survival rates, thyroid cancer can still lead to substantial medical costs and long-term quality-of-life issues for individuals.^[3]^ As a result, thyroid cancer is becoming an increasingly significant public health concern.

Currently, there is widespread Acknowledgments that genetic and environmental factors have considerable impact on progression of thyroid cancer, yet ongoing studies seek to elucidate additional contributing factors.^[4]^ While previous research on thyroid cancer pathogenesis has predominantly concentrated on the pathological alterations in tumor cells, it has often overlooked the crucial role of the tumor microenvironment. Evidence suggests that elements of a persons immune system strongly correlate with thyroid cancer onset, invasion, and metastasis, potentially influencing treatment response and prognosis for patients.^[5]^ Nevertheless, it is still uncertain whether a causal relationship occurs between immune phenotype and thyroid cancer, or if it simply reflects shared risk factors.

The majority of evidence concerning the causal link between potential risk factors and thyroid cancer risk is derived from observational epidemiological studies.^[4]^ However, these studies often face significant limitations, notably due to biases such as confounding variables and the possibility of reverse causation.^[6]^ Confounding occurs when other variables are influencing both the risk factors and the outcome, while reverse causation suggests that the observed relationship might be due to the outcome affecting the risk factors, rather than the other way around. This complexity highlights the need for more robust methodologies to establish causality in thyroid cancer research. Mendelian randomization (MR) is a groundbreaking statistical method designed to overcome the limitations of traditional observational studies. By using genetic variants as proxies, or instrumental variables, for potential risk factors, MR can help isolate causal relationships. This approach allows researchers to evaluate the causal effects within pathways connecting an exposure to an outcome, providing a more robust analysis of the relationship between risk factors and health outcomes.^[7,8]^ In this context, our study employed MR analysis to investigate the causal relationship between 731 immune phenotypes and the risk of thyroid cancer.

## 2. Methods

### 2.1. Study design

**Figure 1.**
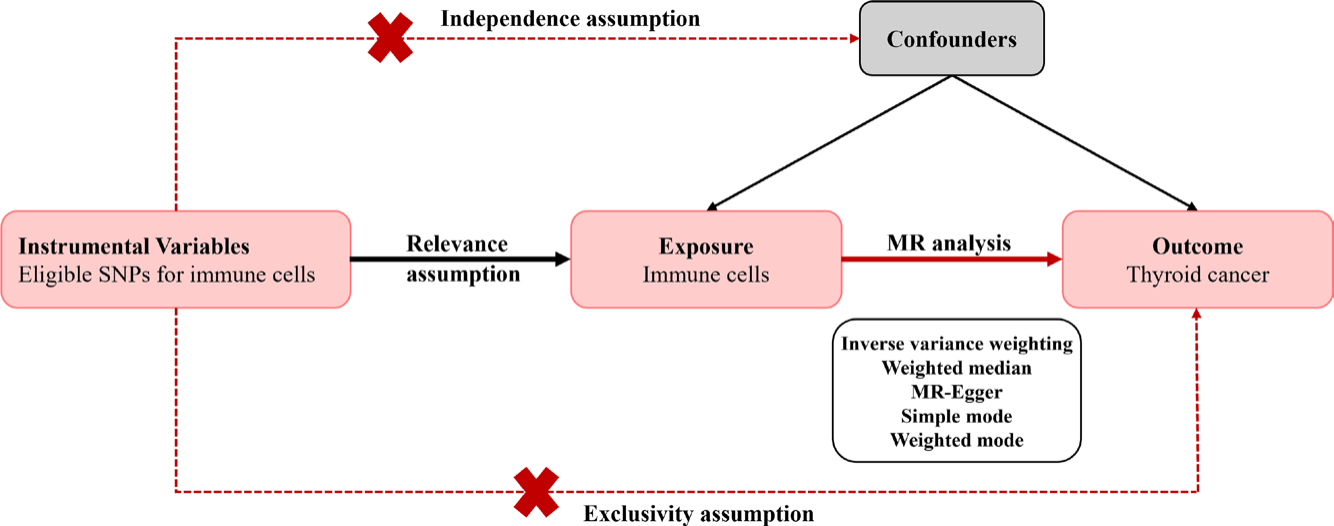
The workflow for our MR analysis.

We investigated the causal relationship between 731 immunocyte phenotypes and thyroid cancer using a MR analysis. In MR analysis, instrumental variables (IVs) are carefully selected based on 3 essential criteria: IVs must be directly associated with the exposure factor; there should be no confounding variables affecting the IVs; and IVs should impact the outcome exclusively through their influence on the exposure factor.^[9,10]^ These criteria are fundamental to ensuring that the selected IVs provide a valid and unbiased estimate of the causal relationship, allowing us to draw robust conclusions from the MR analysis. Data support for this study was provided by the FinnGen and OpenGWAS public databases. Since this study does not involve private information or require institutional informed consent, it does not necessitate ethical review. The workflow for our analysis is depicted in Figure 1.

### 2.2. Collection of genome-wide association studies (GWAS) data

The 731 immune phenotypes were extracted from the GWAS public database, covering the range from GCST90001391 to GCST90002121.^[11]^ Data for thyroid cancer were sourced from the IEU OpenGWAS Project under identifier ebi-a-GCST90018929. In selecting these datasets, we considered several factors, including sample size, publication year, number of single nucleotide polymorphisms (SNPs), and the ethnic background of the subjects.

### 2.3. Selection of IVs

The SNPs used in our study were obtained from GWAS database, with all having a *P* < 1 × 10^‐5^.^[12]^ To avoid bias from linkage disequilibrium, we used an *r*-threshold of 0.001 and set the distance between SNPs at 10,000 kilobases (Kb). Whenever feasible, we replaced missing SNPs in the outcome dataset with those having high linkage disequilibrium (*r*^2^ > 0.8). We aligned alleles across all SNPs in both the exposure and outcome datasets to confirm consistent effect direction.

We used the PhenoScanner V2 database to further confirm that the selected SNP loci did not have other confounding variables.^[13]^ The outcome information was retrieved from the IEU OpenGWAS or FinnGen databases to examine relationships among the SNPs that met our assumptions. Following this, we merged the exposure dataset with the outcome data, removed palindromic sequences, and retained the remaining SNPs as the final IVs for our analysis.

### 2.4. Statistical analysis

All MR analyses in this study were conducted using R version 4.3.1 (The R Foundation for Statistical Computing, Vienna, Austria). We employed a variety of MR analysis methods to explore the causal relationship, including inverse variance weighted (IVW), weighted median, MR-Egger, simple mode, and weighted mode. The IVW method served as our primary approach, providing the main analytical results, while the other methods were used to corroborate the findings through additional supporting analyses.

To evaluate the robustness of our results and to detect potential issues of pleiotropy and heterogeneity, we implemented several sensitivity analyses. Cochran *Q* test was utilized to measure heterogeneity among the IVs, indicating whether there were inconsistencies across the IVs used in the MR analyses. The MR-Egger method, which applies weighted linear regression with an intercept, was used to assess horizontal pleiotropy, providing insights into whether any IVs might influence the outcome through pathways other than the exposure factor.^[14]^

To further examine the stability of our results, we conducted a leave-one-out analysis, which involves removing each SNP at a time to see if any particular SNP had a disproportionately large impact on the overall results. Additionally, scatter plots were created to visually inspect whether outliers were present and if they significantly influenced the outcome. Funnel plots were employed to assess the robustness of the observed correlations and to further check for heterogeneity.

All the results of our MR analyses are reported as odds ratios (ORs) with 95% confidence intervals, providing a statistical measure of the strength and precision of the observed associations. Statistical significance was determined using a threshold of *P* < .05.

## 3. Results

### 3.1. The causal impact of immunocyte on thyroid cancer

**Figure 2.**
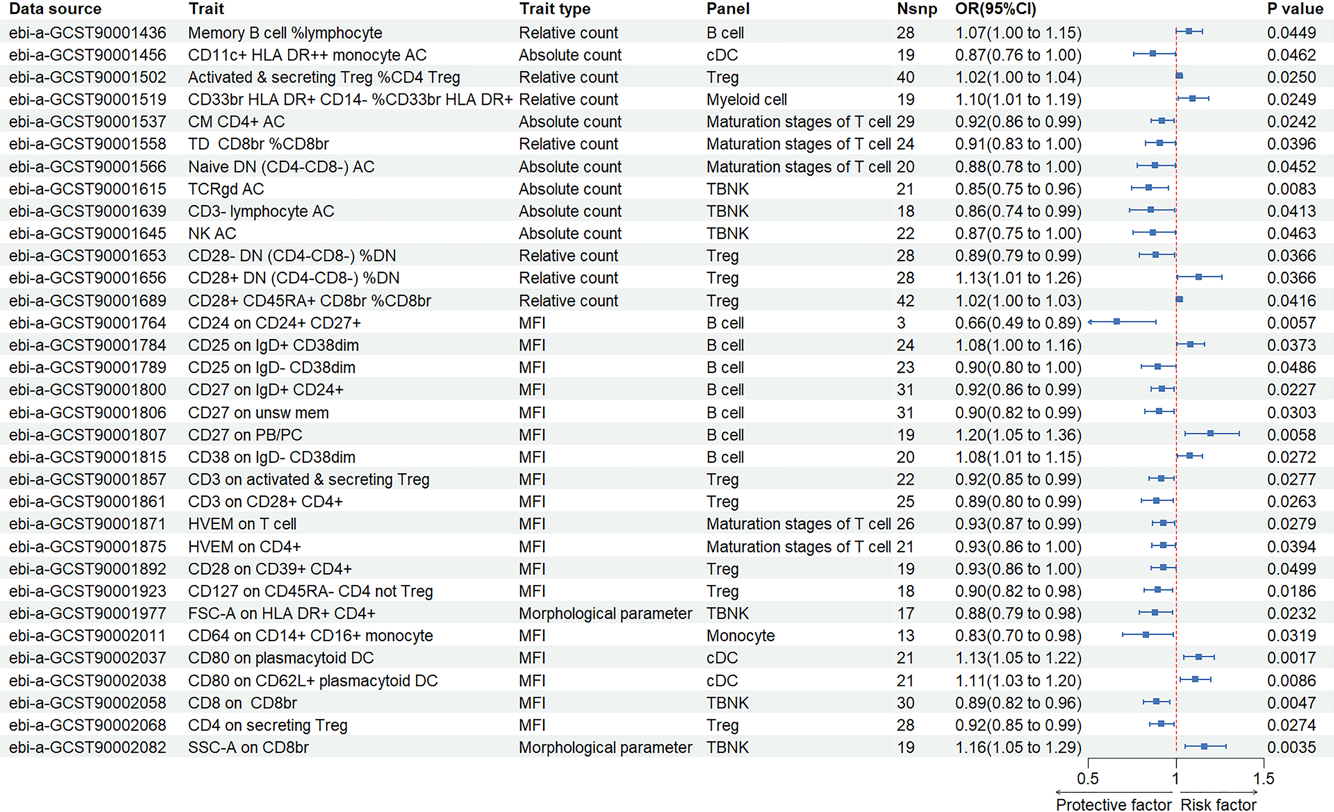
Causal effects of immune cells on thyroid cancer.

**Figure 3.**
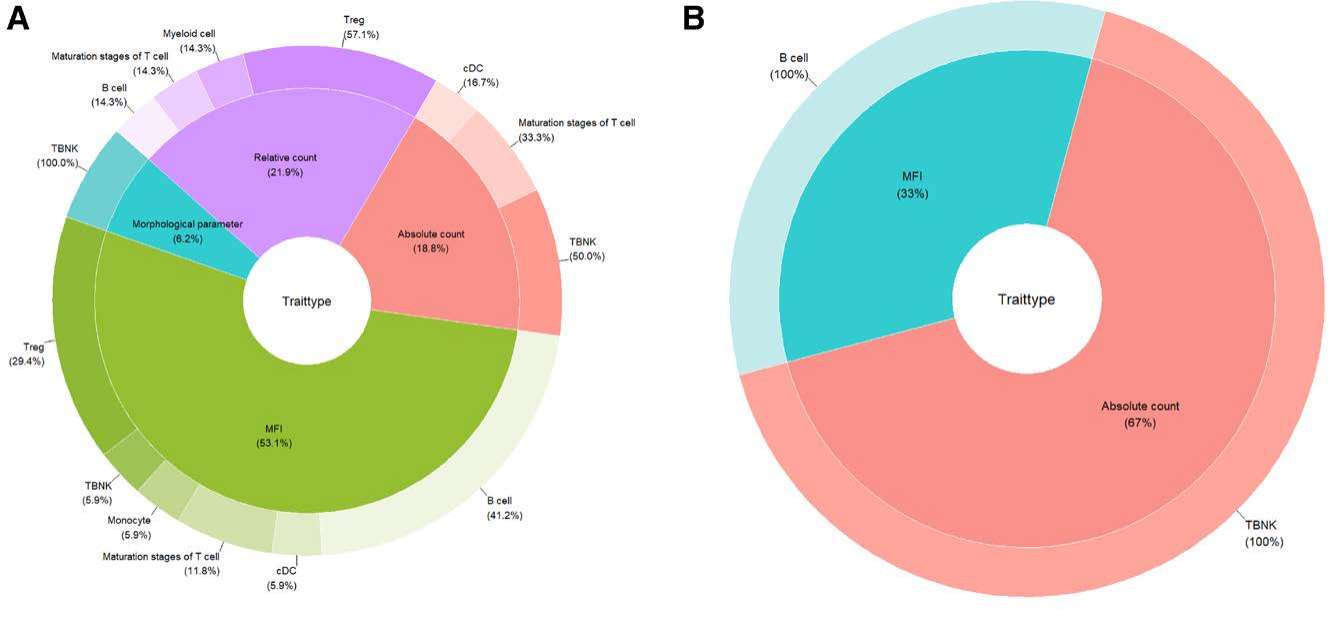
The distribution of immune cells exhibiting significance at a nominal significance level varies across distinct trait categories and diverse analytical panels. (A) The causal effects of immune cell profiles on the risk of thyroid cancer. (B) The causal role of thyroid cancer on immune cells.

The IVW analysis exploring the effects of 7 groups of immune cells on thyroid cancer revealed that 11 immune cell traits had a positive relationship with the occurrence and progression of thyroid cancer (OR > 1, *P* < .05), while 22 traits showed negative correlations (OR < 1, *P* < .05) (Fig. 2, Table S1, Supplemental Digital Content, https://links.lww.com/MD/Q394). These 33 immune cell traits are distributed among several categories: B cells (8 types), regulatory T cells (Tregs, 9 types), conventional dendritic cells (cDCs, 3 types), different maturation stages of T cells (5 types), T, B, and natural killer (TBNK) cells (6 types), myeloid cells (1 type), and monocyte cells (1 type) (Fig. 3A).

The traits positively correlated with thyroid cancer include the following: B cell panel: Memory B cell %lymphocyte, CD25 on IgD+ CD38dim, CD27 on PB/PC, CD38 on IgD‐ CD38dim; Treg panel: Activated and secreting Treg %CD4 Treg, CD28+ DN (CD4‐ CD8‐) %DN, CD28+ CD45RA+ CD8br %CD8br; Myeloid cell panel: CD33br HLA DR+ CD14‐ %CD33br HLA DR+; cDC panel: CD80 on plasmacytoid DC, CD80 on CD62L+ plasmacytoid dendritic cells (DC); TBNK panel: SSC-A on CD8br. The traits negatively correlated with thyroid cancer include: B cell panel: CD24 on CD24+ CD27+, CD25 on IgD‐ CD38dim, CD27 on IgD+ CD24+, CD27 on unswitched memory; Treg panel: CD28‐ DN (CD4‐ CD8‐) %DN, CD3 on activated and secreting Treg, CD3 on CD28+ CD4+, CD28 on CD39+ CD4+, CD127 on CD45RA‐ CD4 not Treg, CD4 on secreting Treg; cDC panel: CD11c+ HLA DR++ monocyte AC; Maturation stages of T cell panel: CM CD4+ AC, TD CD8br %CD8br, Naive DN (CD4‐ CD8‐) AC, HVEM on T cell, HVEM on CD4+; TBNK panel: TCRgd AC, CD3‐ lymphocyte AC, natural killer (NK) AC, FSC-A on HLA DR+ CD4+, CD8 on CD8br; Monocyte panel: CD64 on CD14+ CD16+ monocyte. Further details on the outcomes of the 5 MR analyses conducted can be found in Table S2, Supplemental Digital Content, https://links.lww.com/MD/Q394.

### 3.2. Forward sensitivity analyses

Regarding heterogeneity, the Cochran *Q* test revealed no significant heterogeneity (*P* > .05 for both IVW and MR-Egger methods), and the funnel plot also did not suggest heterogeneity. Concerning pleiotropy, both the MR-Egger and MR-ESTO methods indicated no evidence of horizontal pleiotropy (Table 1). Furthermore, the leave-one-out analysis, funnel plots and scatter plots demonstrated that the results were consistent, providing additional support for the robustness of our findings (Figures S1 and S2, Supplemental Digital Content, https://links.lww.com/MD/Q395).

**Table 1:** Sensitivity analysis of causal effects of immune cells on thyroid cancer.

Immune cell	Cochran *Q* test				MR-Egger intercept test		MR-PRESSO global outlier test	
	MR-Egger		IVW					
	*Q*	*P* value	*Q*	*P* value	Intercept	*P* value	RSSobs	*P* value
Memory B cell %lymphocyte	14.52	.966	15.73	.958	‐0.016	.281	17.10	.962
CD11c+ HLA DR++ monocyte AC	20.35	.256	20.66	.297	0.014	.615	22.59	.334
Activated and secreting Treg %CD4 Treg	32.17	.735	34.75	.664	‐0.023	.117	35.45	.760
CD33brHLA DR+ CD14‐ %CD33br HLA DR+	7.51	.976	7.58	.984	0.006	.797	8.37	.984
CM CD4+ AC	24.78	.587	24.78	.639	‐0.0002	.990	25.64	.712
TD CD8br %CD8br	20.13	.575	20.81	.593	‐0.018	.419	22.14	.661
Naive DN (CD4‐ CD8‐) AC	14.52	.695	14.58	.749	0.006	.809	15.73	.771
TCRgd AC	9.71	.959	9.72	.973	‐0.002	.923	10.44	.983
CD3‐ lymphocyte AC	18.39	.302	18.50	.358	0.009	.755	20.51	.362
NK AC	27.40	.124	28.91	.116	‐0.027	.306	31.51	.125
CD28‐ DN (CD4‐ CD8‐) %DN	34.37	.126	35.07	.137	‐0.016	.471	36.26	.208
CD28+ DN (CD4‐ CD8‐) %DN	34.37	.126	35.07	.137	0.016	.471	36.26	.199
CD28+ CD45RA+ CD8br %CD8br	44.50	.288	44.86	.313	‐0.008	.570	46.45	.359
CD25 on IgD+ CD38dim	20.23	.569	20.34	.621	‐0.007	.742	25.62	.528
CD25 on IgD‐ CD38dim	18.67	.606	19.59	.609	0.021	.349	21.43	.609
CD27 on IgD+ CD24+	30.86	.372	31.48	.392	‐0.012	.453	32.35	.461
CD27 on unsw mem	28.38	.497	30.65	.433	‐0.035	.143	32.61	.469
CD27 on PB/PC	20.26	.261	20.30	.316	0.005	.842	21.86	.402
CD38 on IgD‐ CD38dim	14.11	.722	14.64	.745	0.017	.476	17.63	.735
CD3 on activated and secreting Treg	16.65	.676	19.30	.566	0.035	.119	21.16	.612
CD3 on CD28+ CD4+	24.69	.367	26.40	.333	0.033	.219	28.43	.350
HVEM on T cell	20.01	.696	20.01	.746	0.001	.969	21.44	.786
HVEM on CD4+	10.63	.936	10.66	.955	‐0.004	.872	12.14	.945
CD28 on CD39+ CD4+	9.63	.918	9.73	.940	0.007	.763	10.80	.937
CD127 on CD45RA‐ CD4 not Treg	12.08	.738	12.16	.790	‐0.005	.777	12.76	.857
FSC-A on HLA DR+ CD4+	7.76	.933	8.87	.919	0.024	.310	11.33	.886
CD64 on CD14+ CD16+ monocyte	9.94	.536	11.57	.481	‐0.045	.228	13.10	.549
CD80 on plasmacytoid DC	15.23	.708	16.17	.706	0.017	.345	17.08	.784
CD80 on CD62L+ plasmacytoid DC	16.20	.644	17.82	.599	0.022	.219	19.59	.646
CD8 on CD8br	19.83	.871	19.95	.895	0.006	.732	21.35	.893
CD4 on secreting Treg	23.08	.628	23.22	.673	0.008	.706	24.65	.704
SSC-A on CD8br	17.31	.434	17.59	.483	‐0.011	.605	19.93	.504

DC = dendritic cells, IVW = inverse variance weighting.

### 3.3. Causal effects of thyroid cancer on immunocyte

**Figure 4.**
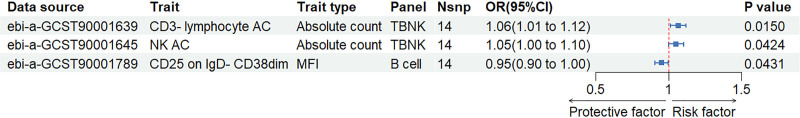
Causal effects of thyroid cancer on immune cells.

The IVW method identified that thyroid cancer was associated with an increase in 2 immune phenotypes and a decrease in 1 immune phenotype (Fig. 4, Table S3, Supplemental Digital Content, https://links.lww.com/MD/Q394). These immune phenotypes are distributed across 2 types of TBNK cells and 1 type of B cell, as depicted in Figure 3B. The traits that showed a positive correlation with thyroid cancer were: TBNK panel: CD3‐ lymphocyte AC, NK AC. The trait that exhibited a negative correlation with thyroid cancer was: B cell panel: CD25 on IgD‐ CD38dim. Additional results from the 5 MR analyses can be found in Table S4, Supplemental Digital Content, https://links.lww.com/MD/Q394.

### 3.4. Reverse sensitivity analyses

The sensitivity analysis results suggest that the 3 immune cell phenotypes identified through the thyroid cancer MR analysis exhibit no significant heterogeneity and horizontal pleiotropy (Cochran *Q* test, MR-Egger, all *P* > .05). These findings support the robustness of the causal estimates (Table 2). Both the leave-one-out analysis, funnel plots and scatter plots reinforce the reliability of the data (Figures S3 and S4, Supplemental Digital Content, https://links.lww.com/MD/Q395).

**Table 2:** Sensitivity analysis of causal effects of thyroid cancer on immune cells.

Immune cell	Cochran *Q* test				MR-Egger intercept test		MR-PRESSO global outlier test	
	MR-Egger		IVW					
	*Q*	*P* value	*Q*	*P* value	Intercept	*P* value	RSSobs	*P* value
CD3‐ lymphocyte AC	4.62	.970	5.87	.951	0.025	.284	6.59	.956
NK AC	8.22	.768	8.71	.795	0.015	.498	10.69	.776
CD25 on IgD‐ CD38dim	7.92	.791	8.69	.796	0.019	.397	10.28	.818

IVW = inverse variance weighting.

## 4. Discussion

This study is the first bidirectional Mendelian randomization analysis that employs 731 immune phenotypes to assess their causal relationship with thyroid cancer. Our analysis identified 33 immune cell phenotypes that may influence the risk of thyroid cancer. Among these, 22 phenotypes are positively linked to an increased risk of thyroid cancer, while 11 show a protective effect against the disease. These findings exclude phenotypes where a reverse causal relationship was observed.

The tumor immune microenvironment represents a vital component of the cellular landscape surrounding tumor cells, exerting a significant influence on tumor development and progression.^[15,16]^ Within the context of thyroid cancer (TC), the tumor immune microenvironment comprises a diverse assortment of immune cells, including TBNK cells, macrophages, and DCs. These immune cells can markedly affect the biological behavior of tumors, with the potential to either suppress or enhance tumor cell proliferation and growth.^[17]^ The dynamic interplay among these immune cell types contributes to the complexity of tumor progression, potentially impacting key processes such as immune surveillance, tumor invasion, metastasis, and response to therapy. Tumor-infiltrating B cells participate in both humoral and cellular immune responses, contributing to a range of immune functions within the tumor microenvironment. However, their specific role in antitumor immunity remains a subject of debate.^[18]^ Although some B cells, particularly those with immunosuppressive subtypes like BCR4 may promote tumor growth,^[19,20]^ B cells also are crucial in fighting tumors. They have the ability to destroy tumor cells through direct lysis in in vitro experiments.^[21]^ Additionally, they can differentiate into plasma cells, which are responsible for producing tumor-specific antibodies. These antibodies can then target and bind to tumor-associated antigens,^[22]^ facilitating the immune system’s ability to recognize and attack tumor cells. In papillary thyroid carcinoma (PTC), the antitumor activity of B cells is closely associated with the presence of tertiary lymphoid structures.^[23]^ Research by Li et al showed a reduction in activated B cells in PTC tissue compared to normal thyroid tissue, suggesting a suppression of humoral immunity.^[24]^ However, this finding contrasts with earlier research by Weber et al, which reported a higher number of CD20+ B cells in PTC compared to normal thyroid tissue, with an even greater difference observed in anaplastic thyroid cancer samples.^[25]^ Our research results also reflect the complexity of B cell functions, as Memory B cell %lymphocyte, CD25 on IgD+ CD38dim, CD27 on PB/PC and CD38 on IgD‐ CD38dim are associated with an increased risk of thyroid cancer, while CD24 on CD24+ CD27+, CD25 on IgD‐ CD38dim, CD27 on IgD+ CD24+ and CD27 on unswitched memory B cells are protective factors against thyroid cancer.

Similarly, Tregs primarily foster disease progression and lymph node metastasis in various tumors by suppressing immune responses, and their presence in PTC is linked to more aggressive disease states.^[26,27]^ Mechanistically, tumors downregulate CD4+ and CD8+ effector T lymphocytes by enhancing the activity of FOXP3+ Tregs, thus facilitating immune evasion.^[28]^ The proportion of CD4+ CD25+ CD127low/‐ Tregs within CD4+ T cells is substantially increased in PTC patients compared to those with multinodular goiter (MNG). FoxP3-positive Tregs demonstrate significant levels of infiltration in both primary PTC and associated metastatic lymph node tissues. In contrast, FoxP3 expression is entirely absent in tissues from MNG.^[29]^ This stark difference in FoxP3+ Treg presence between PTC and MNG may indicate a role for these cells in the tumor’s immunosuppressive microenvironment, potentially contributing to tumor growth and metastasis. Their absence in MNG tissues suggests that FoxP3 expression could serve as a distinguishing factor between benign and malignant thyroid conditions. Our research findings strongly demonstrate that many immune properties against thyroid cancer exist in Treg, such as CD28‐ DN (CD4‐ CD8‐) %DN, CD3 on activated and secreting Treg, CD3 on CD28+ CD4+, CD28 on CD39+ CD4+, CD127 on CD45RA‐ CD4 not Treg and CD4 on secreting Treg. This emphasizes the necessity of further studying the subtle role of Treg cell-related phenotypes in thyroid cancer.

The NK cells are essential to cancer immune surveillance by targeting and destroying cancer cells.^[30,31]^ In PTC, the infiltration of NK cells is greater in during early stages and decreases as the disease progresses, with the density of NK cells showing an inverse correlation with PTC staging.^[32]^ A greater level of NK cell infiltration in patients with PTC has been linked to improved outcomes and a more favorable prognosis.^[33]^ In anaplastic thyroid cancer, cancer cells secrete substantial amounts of the chemokine CXCL10, which serves to attract NK cells due to their expression of the corresponding chemokine receptor, CXCR3.^[34]^ However, despite the presence of NK cells in thyroid cancer tissue, their full antitumor potential often remains unrealized. Our study highlights the role of specific NK cell-related phenotypes in thyroid cancer risk, shedding light on their importance in the tumor immune microenvironment.

In examining the reverse causal relationships between 731 immune cell phenotypes and thyroid cancer, we identified 3 phenotypes with reciprocal associations. This suggests that certain immune cell phenotypes not only influence the development or mitigation of thyroid cancer but can also be affected by its progression. To investigate these potential causal relationships, our study used a bidirectional 2-sample MR approach, which allowed us to explore both directions of causality. To ensure robust causal inference and minimize bias, we employed multiple MR analysis methods while carefully controlling for horizontal pleiotropy and confounding variables.

Our study has several limitations. First, the reliance on data from European cohorts raises questions about the generalizability of our findings to other ethnicities. Second, we used MR analysis with SNP data from the OpenGWAS database, which lacks detailed individual-level information, thereby limiting our ability to categorize thyroid cancer into specific subtypes. Third, the observed effects may not be driven by a single immune phenotype but could result from the interplay of multiple phenotypes, adding variability to the analysis of individual factors. Lastly, we employed a *P*-value threshold of *P* < 1 × 10^‐5^ for selecting IVs, which might suggest a weaker correlation among these IVs. Despite these limitations, our approach offers a broader perspective on the relationship between immune cell phenotypes and thyroid cancer.

## 5. Conclusion

In summary, our study offers new understanding of the immunodynamics of thyroid cancer, laying a cornerstone for future precision medicine. Additionally, this research offers a perspective on the biological mechanisms between TC and immune cells, contributing to the exploration of early intervention and treatment options.

## Author contributions

**Conceptualization:** Tingting Bian, Daishan Jiang, Yifei Liu.

**Data curation:** Tingting Bian, Daishan Jiang.

**Formal analysis:** Daishan Jiang.

**Funding acquisition:** Yifei Liu.

**Investigation:** Yali Zhang, Weiyi Lai, Jianguo Zhang, Yifei Liu.

**Methodology:** Yali Zhang, Weiyi Lai, Jianguo Zhang.

**Project administration:** Jianguo Zhang.

**Resources:** Tingting Bian, Weiyi Lai, Jianguo Zhang.

**Software:** Jianguo Zhang.

**Supervision:** Yali Zhang.

**Validation:** Yali Zhang, Weiyi Lai.

**Visualization:** Weiyi Lai.

**Writing – original draft:** Tingting Bian, Daishan Jiang, Yifei Liu.

**Writing – review & editing:** Tingting Bian, Daishan Jiang, Yifei Liu.

## Supplementary Material




