# Supplementary material for: Investigating the causal relationship between 731 immune phenotypes and thyroid cancer risk: A bidirectional Mendelian randomization study
Source: Medicine (Baltimore). 2025 Oct 17;104(42):e45072. doi: 10.1097/MD.0000000000045072 (PMC12537243; doi:10.1097/MD.0000000000045072)
Supplement: Supplementary file 1 [file medi-104-e45072-s001.pdf]

**Table S1. Analysis for causal associations of immune cells on thyroid cancer risk by IVW method**

| Data source | Trait type | Panel | Statistical trait name | Trait | method | nsnp | b | se | pval | lo_ci | up_ci | or | or_lci95 | or_uci95 |
|---|---|---|---|---|---|---|---|---|---|---|---|---|---|---|
| ebi-a-GCST90001436 | Relative count | B cell | BC_Trait58 | Memory B cell %lymphocyte | Inverse variance weighted | 28 | 0.071471058 | 0.03562825 | 0.044853886 | 0.001639688 | 0.141302427 | 1.074087064 | 1.001641033 | 1.151772922 |
| ebi-a-GCST90001456 | Absolute count | cDC | DC_Trait534 | CD11c+ HLA DR++ monocyte AC | Inverse variance weighted | 19 | -0.139593055 | 0.070037577 | 0.046248685 | -0.276866706 | -0.002319404 | 0.869712088 | 0.758155548 | 0.997683284 |
| ebi-a-GCST90001502 | Relative count | Treg | f_Treg_Trait528 | Activated & secreting Treg %CD4 Treg | Inverse variance weighted | 40 | 0.020152653 | 0.00899205 | 0.0250155 | 0.002528234 | 0.037777072 | 1.020357089 | 1.002531433 | 1.038499696 |
| ebi-a-GCST90001519 | Relative count | Myeloid cell | MDSC_Trait14 | CD33br HLA DR+ CD14- %CD33br HLA DR+ | Inverse variance weighted | 19 | 0.091259681 | 0.040700069 | 0.024945295 | 0.011487547 | 0.171031816 | 1.095553463 | 1.011553782 | 1.186528499 |
| ebi-a-GCST90001537 | Absolute count | Maturation stages of T cell | MT_Trait503 | CM CD4+ AC | Inverse variance weighted | 29 | -0.082693147 | 0.036677381 | 0.024157893 | -0.154580814 | -0.01080548 | 0.920633603 | 0.85677425 | 0.989252689 |
| ebi-a-GCST90001558 | Relative count | Maturation stages of T cell | MT_Trait532 | TD CD8br %CD8br | Inverse variance weighted | 24 | -0.096701758 | 0.04699605 | 0.039623056 | -0.188814016 | -0.0045895 | 0.907826717 | 0.827940476 | 0.995421015 |
| ebi-a-GCST90001566 | Absolute count | Maturation stages of T cell | MT_Trait545 | Naive DN (CD4-CD8-) AC | Inverse variance weighted | 20 | -0.126763407 | 0.063290058 | 0.045188447 | -0.25081192 | -0.002714894 | 0.880942073 | 0.778168716 | 0.997288788 |
| ebi-a-GCST90001615 | Absolute count | TBNK | TB_Trait533 | TCRgd AC | Inverse variance weighted | 21 | -0.166820753 | 0.063219186 | 0.008320795 | -0.290730358 | -0.042911147 | 0.846351304 | 0.747717267 | 0.957996507 |
| ebi-a-GCST90001639 | Absolute count | TBNK | TB_Trait560 | CD3- lymphocyte AC | Inverse variance weighted | 18 | -0.156586843 | 0.076739234 | 0.041299986 | -0.306995742 | -0.006177944 | 0.855057259 | 0.735653733 | 0.9938411 |
| ebi-a-GCST90001645 | Absolute count | TBNK | TB_Trait566 | NK AC | Inverse variance weighted | 22 | -0.142005332 | 0.071262063 | 0.046292127 | -0.281678976 | -0.002331688 | 0.86761663 | 0.754515863 | 0.997671028 |
| ebi-a-GCST90001653 | Relative count | Treg | Treg_Trait505 | CD28- DN (CD4-CD8-) %DN | Inverse variance weighted | 28 | -0.120753678 | 0.057783517 | 0.036639372 | -0.234009373 | -0.007497984 | 0.886252236 | 0.791354399 | 0.992530056 |
| ebi-a-GCST90001656 | Relative count | Treg | Treg_Trait508 | CD28+ DN (CD4-CD8-) %DN | Inverse variance weighted | 28 | 0.120753678 | 0.057783517 | 0.036639372 | 0.007497984 | 0.234009373 | 1.128346942 | 1.007526165 | 1.263656336 |

| | | | | | | | | | | | | | | |
|---|---|---|---|---|---|---|---|---|---|---|---|---|---|---|
| ebi-a-GCST90001689 | Relative count | Treg | Treg_Trait604 | CD28+ CD45RA+ CD8br %CD8br | Inverse variance weighted | 42 | 0.017404792 | 0.008544255 | 0.041648345 | 0.000658052 | 0.034151532 | 1.017557138 | 1.000658269 | 1.034741391 |
| ebi-a-GCST90001764 | MFI | B cell | Bcells.trait418 | CD24 on CD24+ CD27+ | Inverse variance weighted | 3 | -0.413395638 | 0.149462558 | 0.005676926 | -0.706342252 | -0.120449025 | 0.661400556 | 0.493445801 | 0.886522277 |
| ebi-a-GCST90001784 | MFI | B cell | Bcells.trait496 | CD25 on IgD+ CD38dim | Inverse variance weighted | 24 | 0.07738156 | 0.037168026 | 0.037348052 | 0.004532228 | 0.150230891 | 1.080454255 | 1.004542514 | 1.162102531 |
| ebi-a-GCST90001789 | MFI | B cell | Bcells.trait504 | CD25 on IgD- CD38dim | Inverse variance weighted | 23 | -0.109327581 | 0.05543127 | 0.048574365 | -0.21797287 | -0.000682293 | 0.896436714 | 0.804147258 | 0.99931794 |
| ebi-a-GCST90001800 | MFI | B cell | Bcells.trait561 | CD27 on IgD+ CD24+ | Inverse variance weighted | 31 | -0.082822852 | 0.036361751 | 0.022741685 | -0.154091885 | -0.0115382 | 0.9205142 | 0.857193254 | 0.988512669 |
| ebi-a-GCST90001806 | MFI | B cell | Bcells.trait571 | CD27 on unsw mem | Inverse variance weighted | 31 | -0.102629353 | 0.047390024 | 0.0303932 | -0.1955138 | -0.009744905 | 0.902461406 | 0.822411994 | 0.990302423 |
| ebi-a-GCST90001807 | MFI | B cell | Bcells.trait572 | CD27 on PB/PC | Inverse variance weighted | 19 | 0.180822752 | 0.065581635 | 0.005829578 | 0.052282748 | 0.309362757 | 1.198202782 | 1.053673624 | 1.362556557 |
| ebi-a-GCST90001815 | MFI | B cell | Bcells.trait644 | CD38 on IgD- CD38dim | Inverse variance weighted | 20 | 0.075268472 | 0.034080538 | 0.027206159 | 0.008470619 | 0.142066326 | 1.078173572 | 1.008506596 | 1.152653097 |
| ebi-a-GCST90001857 | MFI | Treg | Blue.530.30.Treg.trait15 | CD3 on activated & secreting Treg | Inverse variance weighted | 22 | -0.087573402 | 0.039773672 | 0.027679918 | -0.165529799 | -0.009617004 | 0.916151622 | 0.84744461 | 0.990429091 |
| ebi-a-GCST90001861 | MFI | Treg | Blue.530.30.Treg.trait22 | CD3 on CD28+ CD4+ | Inverse variance weighted | 25 | -0.117668766 | 0.05295226 | 0.02627202 | -0.221455195 | -0.013882336 | 0.888990468 | 0.801351826 | 0.986213579 |
| ebi-a-GCST90001871 | MFI | Maturation stages of T cell | HVEM1000_150630.trait1 | HVEM on T cell | Inverse variance weighted | 26 | -0.076539802 | 0.034822496 | 0.027949198 | -0.144791894 | -0.008287709 | 0.926316045 | 0.865202328 | 0.991746539 |
| ebi-a-GCST90001875 | MFI | Maturation stages of T cell | HVEM1000_150630.trait2 | HVEM on CD4+ | Inverse variance weighted | 21 | -0.074770207 | 0.03628775 | 0.039352612 | -0.145894196 | -0.003646217 | 0.9279567 | 0.864249139 | 0.996360422 |
| ebi-a-GCST90001892 | MFI | Treg | Blue.585.42.Treg.trait19 | CD28 on CD39+ CD4+ | Inverse variance weighted | 19 | -0.075361732 | 0.038427267 | 0.04986123 | -0.150679175 | -4.43E-05 | 0.927407952 | 0.860123604 | 0.999955712 |
| ebi-a-GCST90001923 | MFI | Treg | Blue.670.LP.Treg.trait10 | CD127 on CD45RA- CD4 not Treg | Inverse variance weighted | 18 | -0.108798436 | 0.046246589 | 0.018644073 | -0.199441749 | -0.018155122 | 0.896911185 | 0.819187938 | 0.982008689 |
| ebi-a-GCST9000 | Morphological parameter | TBNK | FSC.TBNK.trait8 | FSC-A on HLA DR+ CD4+ | Inverse variance | 17 | -0.1252 | 0.055151328 | 0.02318618 | -0.2333 | -0.0171 | 0.882308971 | 0.791908376 | 0.983029278 |

| | | | | | | | | | | | | | |
|---|---|---|---|---|---|---|---|---|---|---|---|---|---|
| 1977 | | | | | weighted | | 12977 | | | 0958 | 16374 | | | |
| ebi-a-GCST90002011 | MFI | Monocyte | mono.trait6 | CD64 on CD14+ CD16+ monocyte | Inverse variance weighted | 13 | -0.188929959 | 0.088037973 | 0.031872553 | -0.361484385 | -0.016375533 | 0.827844488 | 0.696641474 | 0.983757817 |
| ebi-a-GCST90002037 | MFI | cDC | Red.780.60.cDC.trait3 | CD80 on plasmacytoid DC | Inverse variance weighted | 21 | 0.12072165 | 0.038536495 | 0.001732313 | 0.045190119 | 0.196253181 | 1.128310803 | 1.046226748 | 1.216834946 |
| ebi-a-GCST90002038 | MFI | cDC | Red.780.60.cDC.trait4 | CD80 on CD62L+ plasmacytoid DC | Inverse variance weighted | 21 | 0.103576151 | 0.03942255 | 0.008605717 | 0.026307953 | 0.180844349 | 1.109130251 | 1.026657062 | 1.19822866 |
| ebi-a-GCST90002058 | MFI | TBNK | Red.780.60.TBNK.trait1 | CD8 on  CD8br | Inverse variance weighted | 30 | -0.120217769 | 0.042497088 | 0.004671599 | -0.203512061 | -0.036923478 | 0.886727314 | 0.815860364 | 0.963749881 |
| ebi-a-GCST90002068 | MFI | Treg | Red.780.60.Treg.trait7 | CD4 on secreting Treg | Inverse variance weighted | 28 | -0.086557134 | 0.039234947 | 0.027375363 | -0.163457631 | -0.009656637 | 0.91708315 | 0.849202478 | 0.990389838 |
| ebi-a-GCST90002082 | Morphological parameter | TBNK | SSC.TBNK.trait5 | SSC-A on CD8br | Inverse variance weighted | 19 | 0.150288784 | 0.051436625 | 0.003479877 | 0.049472999 | 0.251104569 | 1.16216981 | 1.050717221 | 1.285444495 |

| Table S2. Analysis for causal associations of immune cells on thyroid cancer risk by five MR methods | | | | | | | | | | | | | | |
|---|---|---|---|---|---|---|---|---|---|---|---|---|---|---|
| Data source | Trait type | Panel | Statistical trait name | Trait | method | nsnp | b | se | pval | lo_ci | up_ci | or | or_lci95 | or_uci95 |
| ebi-a-GCST90001436 | Relative count | B cell | BC_Trait58 | Memory B cell %lymphocyte | MR Egger | 28 | 0.098872115 | 0.043458062 | 0.031381675 | 0.013694313 | 0.184049917 | 1.103925115 | 1.01378851 | 1.202075826 |
| | | | | | Weighted median | 28 | 0.070860493 | 0.049207464 | 0.149857374 | -0.025586138 | 0.167307123 | 1.073431464 | 0.974738414 | 1.182117265 |
| | | | | | Inverse variance weighted | 28 | 0.071471058 | 0.03562825 | 0.044853886 | 0.001639688 | 0.141302427 | 1.074087064 | 1.001641033 | 1.151772922 |
| | | | | | Simple mode | 28 | 0.130054214 | 0.10988842 | 0.246922356 | -0.085327088 | 0.345435517 | 1.138890126 | 0.918211899 | 1.412604999 |
| | | | | | Weighted mode | 28 | 0.080735651 | 0.045184176 | 0.085201677 | -0.007825333 | 0.169296635 | 1.084084282 | 0.992205205 | 1.184471443 |
| ebi-a- | Absolute | cDC | DC_Trait534 | CD11c+ | MR | 19 | - | 0.11533 | 0.12525 | - | 0.04007 | 0.83028 | 0.66229 | 1.04089 |

| | | | | | | | | | | | | | |
|---|---|---|---|---|---|---|---|---|---|---|---|---|---|
| GCST90001456 | count | | | HLA DR++ monocyte AC | Egger | | 0.185983982 | 817 | 9287 | 0.412046794 | 8831 | 6895 | 3284 | 2826 |
| | | | | | Weighted median | 19 | -0.186807209 | 0.095323008 | 0.05002754 | -0.373640304 | 2.59E-05 | 0.829603661 | 0.688224419 | 1.000025887 |
| | | | | | Inverse variance weighted | 19 | -0.139593055 | 0.070037577 | 0.046248685 | -0.276866706 | -0.002319404 | 0.869712088 | 0.758155548 | 0.997683284 |
| | | | | | Simple mode | 19 | -0.123212044 | 0.14953498 | 0.420742048 | -0.416300605 | 0.169876517 | 0.88407618 | 0.659481997 | 1.185158496 |
| | | | | | Weighted mode | 19 | -0.180781501 | 0.109788042 | 0.116978903 | -0.395966063 | 0.034403061 | 0.834617702 | 0.673029536 | 1.035001691 |
| ebi-a-GCST90001502 | Relative count | Treg | f_Treg_Trait528 | Activated & secreting Treg %CD4 Treg | MR Egger | 40 | 0.027021635 | 0.009959333 | 0.009958785 | 0.007501342 | 0.046541928 | 1.02739003 | 1.007529548 | 1.047642004 |
| | | | | | Weighted | 40 | 0.021971801 | 0.01245791 | 0.077785405 | -0.00244 | 0.046389304 | 1.022214958 | 0.997557286 | 1.04748212 |

| | | | | | | | | | | | | | | |
|---|---|---|---|---|---|---|---|---|---|---|---|---|---|---|
| | | | | | median | | | | | 5702 | | | | |
| | | | | | Inverse variance weighted | 40 | 0.020152653 | 0.00899205 | 0.0250155 | 0.002528234 | 0.037777072 | 1.020357089 | 1.002531433 | 1.038499696 |
| | | | | | Simple mode | 40 | 6.56E-05 | 0.03448282 | 0.998491702 | -0.067520723 | 0.067651932 | 1.000065607 | 0.93470835 | 1.069992814 |
| | | | | | Weighted mode | 40 | 0.019924912 | 0.010506125 | 0.065318306 | -0.000667093 | 0.040516918 | 1.020124739 | 0.999333129 | 1.041348927 |
| ebi-a-GCST90001519 | Relative count | Myeloid cell | MDSC_Trait14 | CD33br HLA DR+ CD14- %CD33br HLA DR+ | MR Egger | 19 | 0.078179342 | 0.064539025 | 0.242331694 | -0.048317146 | 0.20467583 | 1.081316567 | 0.952831552 | 1.227127203 |
| | | | | | Weighted median | 19 | 0.072373503 | 0.054841621 | 0.186941192 | -0.035116073 | 0.179863079 | 1.075056806 | 0.965493342 | 1.197053451 |
| | | | | | Inverse varia | 19 | 0.091259681 | 0.040700069 | 0.024945295 | 0.011487547 | 0.171031816 | 1.095553463 | 1.011553782 | 1.186528499 |

| | | | | | | | | | | | | | | |
|---|---|---|---|---|---|---|---|---|---|---|---|---|---|---|
| | | | | | nce weighted | | | | | | | | | |
| | | | | | Simple mode | 19 | 0.027803859 | 0.082599879 | 0.740304343 | -0.134091903 | 0.189699622 | 1.028193994 | 0.87450969 | 1.20888642 |
| | | | | | Weighted mode | 19 | 0.068435786 | 0.061692451 | 0.28189968 | -0.052481418 | 0.18935299 | 1.07083186 | 0.948871953 | 1.208467454 |
| ebi-a-GCST90001537 | Absolute count | Maturation stages of T cell | MT_Trait503 | CM CD4+ AC | MR Egger | 29 | -0.08230604 | 0.04874534 | 0.102835961 | -0.177846906 | 0.013234826 | 0.920990056 | 0.837070564 | 1.013322794 |
| | | | | | Weighted median | 29 | -0.081157726 | 0.054972926 | 0.139857535 | -0.18890466 | 0.026589208 | 0.922048249 | 0.827865431 | 1.026945855 |
| | | | | | Inverse variance weighted | 29 | -0.082693147 | 0.036677381 | 0.024157893 | -0.154580814 | -0.01080548 | 0.920633603 | 0.85677425 | 0.989252689 |
| | | | | | Simple | 29 | -0.13291 | 0.102181966 | 0.203930332 | -0.33319 | 0.067358146 | 0.875536438 | 0.716630321 | 1.069678511 |

| | | | | | | | | | | | | | | |
|---|---|---|---|---|---|---|---|---|---|---|---|---|---|---|
| | | | | | mode | | 8508 | | | 5163 | | | | |
| | | | | | Weighted mode | 29 | -0.08606922 | 0.047452538 | 0.080440566 | -0.179076195 | 0.006937755 | 0.917530717 | 0.836042195 | 1.006961877 |
| ebi-a-GCST90001558 | Relative count | Maturation stages of T cell | MT_Trait532 | TD CD8br %CD8br | MR Egger | 24 | -0.051351217 | 0.072392977 | 0.485564005 | -0.193241453 | 0.090539018 | 0.949944975 | 0.824282924 | 1.094764223 |
| | | | | | Weighted median | 24 | -0.05171272 | 0.065491869 | 0.429758372 | -0.180076784 | 0.076651344 | 0.949601629 | 0.835206078 | 1.079665578 |
| | | | | | Inverse variance weighted | 24 | -0.096701758 | 0.04699605 | 0.039623056 | -0.188814016 | -0.0045895 | 0.907826717 | 0.827940476 | 0.995421015 |
| | | | | | Simple mode | 24 | -0.096987206 | 0.101220737 | 0.347938988 | -0.29537985 | 0.101405438 | 0.907567617 | 0.744248831 | 1.106725259 |
| | | | | | Weighted mode | 24 | -0.059555404 | 0.06065629 | 0.336389654 | -0.178441732 | 0.059330925 | 0.942183332 | 0.836572801 | 1.061126336 |
| ebi-a- | Absolute | Matur | MT_Trait545 | Naive DN | MR | 20 | - | 0.10362 | 0.17338 | - | 0.05620 | 0.86337 | 0.70467 | 1.05781 |

| | | | | | | | | | | | | | | |
|---|---|---|---|---|---|---|---|---|---|---|---|---|---|---|
| GCST90001566 | count | ation stages of T cell | | (CD4-CD8-) AC | Egger | | 0.146902739 | 8656 | 9776 | 0.350014905 | 9428 | 7947 | 7586 | 9197 |
| | | | | | Weighted median | 20 | -0.160862108 | 0.09200249 | 0.08038547 | -0.34118699 | 0.019462773 | 0.851409465 | 0.71092596 | 1.019653407 |
| | | | | | Inverse variance weighted | 20 | -0.126763407 | 0.063290058 | 0.045188447 | -0.25081192 | -0.002714894 | 0.880942073 | 0.778168716 | 0.997288788 |
| | | | | | Simple mode | 20 | -0.14853728 | 0.143771144 | 0.314505538 | -0.430328721 | 0.133254161 | 0.861967872 | 0.650295294 | 1.142540351 |
| | | | | | Weighted mode | 20 | -0.176173086 | 0.092462804 | 0.071983281 | -0.357400183 | 0.00505401 | 0.838472842 | 0.699492517 | 1.005066803 |
| ebi-a-GCST90001615 | Absolute count | TBNK | TB_Trait533 | TCRgd AC | MR Egger | 21 | -0.159178682 | 0.100653985 | 0.13028042 | -0.356460492 | 0.038103128 | 0.852843958 | 0.700150133 | 1.038838361 |
| | | | | | Weighted media | 21 | -0.185514452 | 0.085419661 | 0.029870724 | -0.352936988 | -0.018091915 | 0.830676831 | 0.702621466 | 0.982070761 |

| | | | | | | | | | | | | | | |
|---|---|---|---|---|---|---|---|---|---|---|---|---|---|---|
| | | | | | n | | | | | | | | | |
| | | | | | Inverse variance weighted | 21 | -0.166820753 | 0.063219186 | 0.008320795 | -0.290730358 | -0.042911147 | 0.846351304 | 0.747717267 | 0.957996507 |
| | | | | | Simple mode | 21 | -0.195639744 | 0.14372381 | 0.188584881 | -0.477338411 | 0.086058923 | 0.822308423 | 0.620432532 | 1.089870545 |
| | | | | | Weighted mode | 21 | -0.175862898 | 0.091824639 | 0.069877353 | -0.35583919 | 0.004113394 | 0.838732968 | 0.700585273 | 1.004121866 |
| ebi-a-GCST90001639 | Absolute count | TBNK | TB_Trait560 | CD3-lymphocyte AC | MR Egger | 18 | -0.197264199 | 0.1502469 | 0.207725453 | -0.491748123 | 0.097219725 | 0.820973704 | 0.611556384 | 1.102102507 |
| | | | | | Weighted median | 18 | -0.184822829 | 0.10865238 | 0.088934122 | -0.397781494 | 0.028135837 | 0.831251545 | 0.671808806 | 1.028535388 |
| | | | | | Inverse variance weighted | 18 | -0.156586843 | 0.076739234 | 0.041299986 | -0.306995742 | -0.006177944 | 0.855057259 | 0.735653733 | 0.9938411 |

| | | | | | | | | | | | | | | |
|---|---|---|---|---|---|---|---|---|---|---|---|---|---|---|
| | | | | | Simple mode | 18 | -0.216699182 | 0.180323414 | 0.245944185 | -0.570133073 | 0.136734709 | 0.805172143 | 0.565450188 | 1.146523946 |
| | | | | | Weighted mode | 18 | -0.178136553 | 0.13288517 | 0.19771372 | -0.438591487 | 0.08231838 | 0.836828144 | 0.644944194 | 1.085801452 |
| ebi-a-GCST90001645 | Absolute count | TBNK | TB_Trait566 | NK AC | MR Egger | 22 | -0.032061394 | 0.126548415 | 0.802581672 | -0.280096287 | 0.215973499 | 0.968447124 | 0.755710973 | 1.241069489 |
| | | | | | Weighted median | 22 | -0.083443797 | 0.099165921 | 0.40009231 | -0.277809002 | 0.110921408 | 0.919942789 | 0.757441478 | 1.117307092 |
| | | | | | Inverse variance weighted | 22 | -0.142005332 | 0.071262063 | 0.046292127 | -0.281678976 | -0.002331688 | 0.86761663 | 0.754515863 | 0.997671028 |
| | | | | | Simple mode | 22 | -0.412429531 | 0.19401167 | 0.045547642 | -0.792692406 | -0.032166657 | 0.662039848 | 0.452624505 | 0.968345187 |
| | | | | | Weighted mode | 22 | -0.088333274 | 0.10068429 | 0.390234609 | -0.285674483 | 0.109007934 | 0.915455728 | 0.751507204 | 1.115171199 |
| ebi-a- | Relative | Treg | Treg_Trait505 | CD28- DN | MR | 28 | - | 0.09122 | 0.45325 | - | 0.10934 | 0.93289 | 0.78015 | 1.11554 |

| | | | | | | | | | | | | |
|---|---|---|---|---|---|---|---|---|---|---|---|---|
| GCST90001653 | count | | | (CD4-CD8-) %DN | Egger | | 0.069460457 | 536 | 9986 | 0.248262163 | 1248 | 7022 | 5389 | 2962 |
| | | | | | Weighted median | 28 | -0.087037837 | 0.075647026 | 0.249905709 | -0.235306009 | 0.061230335 | 0.916642412 | 0.790328965 | 1.063143765 |
| | | | | | Inverse variance weighted | 28 | -0.120753678 | 0.057783517 | 0.036639372 | -0.234009373 | -0.007497984 | 0.886252236 | 0.791354399 | 0.992530056 |
| | | | | | Simple mode | 28 | -0.04496713 | 0.111734579 | 0.690524423 | -0.263966905 | 0.174032646 | 0.956028906 | 0.767998956 | 1.190094417 |
| | | | | | Weighted mode | 28 | -0.08028493 | 0.067671777 | 0.245804435 | -0.212921613 | 0.052351754 | 0.922853361 | 0.808219489 | 1.053746336 |
| ebi-a-GCST90001656 | Relative count | Treg | Treg_Trait508 | CD28+ DN (CD4-CD8-) %DN | MR Egger | 28 | 0.069460457 | 0.09122536 | 0.453259986 | -0.109341248 | 0.248262163 | 1.071929674 | 0.896424462 | 1.281795928 |
| | | | | | Weighted media | 28 | 0.087037837 | 0.082767727 | 0.292987015 | -0.075186908 | 0.249262582 | 1.090937957 | 0.9275701 | 1.283078903 |

| | | | | | | | | | | | | | | |
|---|---|---|---|---|---|---|---|---|---|---|---|---|---|---|
| | | | | | n | | | | | | | | | |
| | | | | | Inverse variance weighted | 28 | 0.120753678 | 0.057783517 | 0.036639372 | 0.007497984 | 0.234009373 | 1.128346942 | 1.007526165 | 1.263656336 |
| | | | | | Simple mode | 28 | 0.04496713 | 0.109341902 | 0.684133515 | -0.169342998 | 0.259277258 | 1.045993477 | 0.844219288 | 1.295993079 |
| | | | | | Weighted mode | 28 | 0.08028493 | 0.069010593 | 0.254857628 | -0.054975833 | 0.215545692 | 1.083595772 | 0.946508022 | 1.240538664 |
| ebi-a-GCST90001689 | Relative count | Treg | Treg_Trait604 | CD28+ CD45RA+ CD8br %CD8br | MR Egger | 42 | 0.019011433 | 0.009060552 | 0.042243566 | 0.001252751 | 0.036770115 | 1.019193301 | 1.001253536 | 1.037454498 |
| | | | | | Weighted median | 42 | 0.011586107 | 0.012011236 | 0.334742326 | -0.011955916 | 0.03512813 | 1.011653486 | 0.988115272 | 1.035752411 |
| | | | | | Inverse variance weigh | 42 | 0.017404792 | 0.008544255 | 0.041648345 | 0.000658052 | 0.034151532 | 1.017557138 | 1.000658269 | 1.034741391 |

| | | | | | | | | | | | | | | |
|---|---|---|---|---|---|---|---|---|---|---|---|---|---|---|
| | | | | | ted | | | | | | | | | |
| | | | | | Simple mode | 42 | 0.011436653 | 0.023793961 | 0.633317668 | -0.035199511 | 0.058072818 | 1.011502302 | 0.965412786 | 1.059792164 |
| | | | | | Weighted mode | 42 | 0.015859467 | 0.008831925 | 0.079915081 | -0.001451106 | 0.03317004 | 1.015985896 | 0.998549946 | 1.033726299 |
| ebi-a-GCST90001764 | MFI | B cell | Bcells.trait418 | CD24 on CD24+ CD27+ | MR Egger | 3 | -0.438199937 | 0.284350964 | 0.366442417 | -0.995527826 | 0.119127951 | 0.645196771 | 0.369528346 | 1.126514048 |
| | | | | | Weighted median | 3 | -0.426919416 | 0.211044265 | 0.043084449 | -0.840566175 | -0.013272658 | 0.652516132 | 0.431466169 | 0.986815036 |
| | | | | | Inverse variance weighted | 3 | -0.413395638 | 0.149462558 | 0.005676926 | -0.706342252 | -0.120449025 | 0.661400556 | 0.493445801 | 0.886522277 |
| | | | | | Simple mode | 3 | -0.48884666 | 0.260004797 | 0.2008383 | -0.998456062 | 0.020762741 | 0.613333368 | 0.368447863 | 1.020979786 |
| | | | | | Weighted mode | 3 | -0.44431097 | 0.214524555 | 0.174157169 | -0.864779097 | -0.023842843 | 0.641265975 | 0.421144574 | 0.976439152 |

| | | | | | | | | | | | | | | |
|---|---|---|---|---|---|---|---|---|---|---|---|---|---|---|
| ebi-a-GCST90001784 | MFI | B cell | Bcells.trait496 | CD25 on IgD+ CD38dim | MR Egger | 24 | 0.086868392 | 0.046824245 | 0.077017325 | -0.004907127 | 0.178643912 | 1.090753119 | 0.995104893 | 1.195594931 |
| | | | | | Weighted median | 24 | 0.071833501 | 0.053400274 | 0.178563965 | -0.032831036 | 0.176498039 | 1.07447643 | 0.967702053 | 1.193032087 |
| | | | | | Inverse variance weighted | 24 | 0.07738156 | 0.037168026 | 0.037348052 | 0.004532228 | 0.150230891 | 1.080454255 | 1.004542514 | 1.162102531 |
| | | | | | Simple mode | 24 | 0.074060199 | 0.092508376 | 0.431568229 | -0.107256218 | 0.255376615 | 1.07687163 | 0.898295484 | 1.29094772 |
| | | | | | Weighted mode | 24 | 0.09647176 | 0.058861937 | 0.114832296 | -0.018897637 | 0.211841157 | 1.101278481 | 0.981279804 | 1.235951548 |
| ebi-a-GCST90001789 | MFI | B cell | Bcells.trait504 | CD25 on IgD- CD38dim | MR Egger | 23 | -0.176371758 | 0.089323647 | 0.061617915 | -0.351446106 | -0.00129741 | 0.838306278 | 0.703669772 | 0.998703432 |
| | | | | | Weighted median | 23 | -0.19886823 | 0.081853482 | 0.015117018 | -0.359301055 | -0.038435405 | 0.819657892 | 0.698164134 | 0.962293862 |

| | | | | | | | | | | | | | | |
|---|---|---|---|---|---|---|---|---|---|---|---|---|---|---|
| | | | | | Inverse variance weighted | 23 | -0.109327581 | 0.05543127 | 0.048574365 | -0.21797287 | -0.000682293 | 0.896436714 | 0.804147258 | 0.99931794 |
| | | | | | Simple mode | 23 | -0.269296524 | 0.152473186 | 0.091238779 | -0.568143969 | 0.029550921 | 0.763916702 | 0.566576046 | 1.029991882 |
| | | | | | Weighted mode | 23 | -0.20985377 | 0.090919749 | 0.030775334 | -0.388056478 | -0.031651062 | 0.810702786 | 0.678374029 | 0.96884459 |
| ebi-a-GCST90001800 | MFI | B cell | Bcells.trait561 | CD27 on IgD+ CD24+ | MR Egger | 31 | -0.054617018 | 0.052124404 | 0.303376156 | -0.15678085 | 0.047546813 | 0.946847704 | 0.854891388 | 1.048695293 |
| | | | | | Weighted median | 31 | -0.08241778 | 0.050648145 | 0.103681556 | -0.181688145 | 0.016852584 | 0.92088715 | 0.833861344 | 1.01699539 |
| | | | | | Inverse variance weighted | 31 | -0.082822852 | 0.036361751 | 0.022741685 | -0.154091885 | -0.01155382 | 0.9205142 | 0.857193254 | 0.988512669 |
| | | | | | Simpl | 31 | - | 0.08695 | 0.45146 | - | 0.10409 | 0.93581 | 0.78916 | 1.10970 |

| | | | | | | | | | | | | | |
|---|---|---|---|---|---|---|---|---|---|---|---|---|---|
| | | | | | e mode | | 0.06634236 | 7952 | 6185 | 0.236779946 | 5226 | 0426 | 4928 | 6123 |
| | | | | | Weighted mode | 31 | -0.083433848 | 0.046299254 | 0.081593765 | -0.174180385 | 0.00731269 | 0.919951942 | 0.840145334 | 1.007339493 |
| ebi-a-GCST90001806 | MFI | B cell | Bcells.trait571 | CD27 on unsw mem | MR Egger | 31 | 0.016089244 | 0.091701612 | 0.861943723 | -0.163645916 | 0.195824404 | 1.016219373 | 0.849042601 | 1.216313307 |
| | | | | | Weighted median | 31 | -0.045580195 | 0.07131697 | 0.522744025 | -0.185361456 | 0.094201067 | 0.955442978 | 0.830803931 | 1.098780652 |
| | | | | | Inverse variance weighted | 31 | -0.102629353 | 0.047390024 | 0.03033932 | -0.1955138 | -0.009744905 | 0.902461406 | 0.822411994 | 0.990302423 |
| | | | | | Simple mode | 31 | -0.031913071 | 0.126308026 | 0.802253644 | -0.279476802 | 0.21565066 | 0.968590777 | 0.75617927 | 1.240668888 |
| | | | | | Weighted mode | 31 | -0.02777793 | 0.074511919 | 0.711919479 | -0.17382129 | 0.118265431 | 0.972604329 | 0.84044708 | 1.125542825 |
| ebi-a-GCST900 | MFI | B cell | Bcells.trait572 | CD27 on PB/PC | MR Egger | 19 | 0.167981077 | 0.092687664 | 0.087634378 | -0.01368 | 0.349648899 | 1.182914226 | 0.986406492 | 1.4185694 |

| | | | | | | | | | | | | | | |
|---|---|---|---|---|---|---|---|---|---|---|---|---|---|---|
| 01807 | | | | | | | | | | 6746 | | | | |
| | | | | | Weighted median | 19 | 0.221837226 | 0.098010134 | 0.023610359 | 0.029737363 | 0.413937088 | 1.248368159 | 1.030183934 | 1.512761953 |
| | | | | | Inverse variance weighted | 19 | 0.180822752 | 0.065581635 | 0.005829578 | 0.052282748 | 0.309362757 | 1.198202782 | 1.053673624 | 1.362556557 |
| | | | | | Simple mode | 19 | 0.182136007 | 0.143539556 | 0.220643876 | -0.099201523 | 0.463473537 | 1.199777361 | 0.905560198 | 1.589585892 |
| | | | | | Weighted mode | 19 | 0.182136007 | 0.085262713 | 0.046652903 | 0.01502109 | 0.349250924 | 1.199777361 | 1.015134473 | 1.418004957 |
| ebi-a-GCST90001815 | MFI | B cell | Bcells.trait644 | CD38 on IgD- CD38dim | MR Egger | 20 | 0.05912575 | 0.04066845 | 0.163202753 | -0.020584412 | 0.138835912 | 1.060908641 | 0.979626001 | 1.148935557 |
| | | | | | Weighted median | 20 | 0.040732699 | 0.052671867 | 0.439327503 | -0.062504159 | 0.143969558 | 1.041573655 | 0.939409155 | 1.154848952 |
| | | | | | Inverse se | 20 | 0.075268472 | 0.034080538 | 0.027206159 | 0.008470619 | 0.142066326 | 1.078173572 | 1.008506596 | 1.152653097 |

| | | | | | | | | | | | | | | |
|---|---|---|---|---|---|---|---|---|---|---|---|---|---|---|
| | | | | | variance weighted | | | | | | | | | |
| | | | | | Simple mode | 20 | 0.03327946 | 0.085634167 | 0.701875141 | -0.134563507 | 0.201122427 | 1.033839416 | 0.874097366 | 1.222774463 |
| | | | | | Weighted mode | 20 | 0.071662054 | 0.043685447 | 0.117370882 | -0.013961423 | 0.15728553 | 1.074292229 | 0.986135586 | 1.17032973 |
| ebi-a-GCST90001857 | MFI | Treg | Blue.530.30.Treg.trait15 | CD3 on activated & secreting Treg | MR Egger | 22 | -0.169160692 | 0.063915905 | 0.015479971 | -0.294435865 | -0.043885519 | 0.844373208 | 0.744951722 | 0.957063517 |
| | | | | | Weighted median | 22 | -0.14331285 | 0.056449547 | 0.011124049 | -0.253953962 | -0.032671738 | 0.866482947 | 0.775727514 | 0.967856218 |
| | | | | | Inverse variance weighted | 22 | -0.087573402 | 0.039773672 | 0.027679918 | -0.165529799 | -0.009617004 | 0.916151622 | 0.84744461 | 0.990429091 |
| | | | | | Simple | 22 | -0.12985 | 0.090418403 | 0.165695264 | -0.30707 | 0.047367348 | 0.878224764 | 0.735597053 | 1.048507105 |

| | | | | | | | | | | | | | | |
|---|---|---|---|---|---|---|---|---|---|---|---|---|---|---|
| | | | | | mode | | 2722 | | | 2793 | | | | |
| | | | | | Weighted mode | 22 | -0.139466473 | 0.052653078 | 0.015017345 | -0.242666507 | -0.03626644 | 0.869822185 | 0.784533106 | 0.964383309 |
| ebi-a-GCST90001861 | MFI | Treg | Blue.530.30.Treg.trait22 | CD3 on CD28+ CD4+ | MR Egger | 25 | -0.223018185 | 0.098336793 | 0.033039783 | -0.4157583 | -0.03027807 | 0.800100299 | 0.659839735 | 0.970175719 |
| | | | | | Weighted median | 25 | -0.149571943 | 0.076412829 | 0.050298195 | -0.299341088 | 0.000197201 | 0.861076487 | 0.741306516 | 1.000197221 |
| | | | | | Inverse variance weighted | 25 | -0.117668766 | 0.05295226 | 0.02627202 | -0.221455195 | -0.013882336 | 0.888990468 | 0.801351826 | 0.986213579 |
| | | | | | Simple mode | 25 | -0.290755523 | 0.13502561 | 0.041553607 | -0.55540572 | -0.026105326 | 0.747698451 | 0.573839396 | 0.974232472 |
| | | | | | Weighted mode | 25 | -0.174666112 | 0.081274868 | 0.041924582 | -0.333964854 | -0.015367371 | 0.839737352 | 0.716078949 | 0.984750105 |
| ebi-a-GCST90001871 | MFI | Maturation stages | HVEM1000_150630.trait1 | HVEM on T cell | MR Egger | 26 | -0.078565087 | 0.062745744 | 0.222592167 | -0.201546745 | 0.04441657 | 0.924441889 | 0.817465365 | 1.045417754 |

| | | | | | | | | | | | | | | |
|---|---|---|---|---|---|---|---|---|---|---|---|---|---|---|
| | | of T cell | | | | | | | | | | | | |
| | | | | | Weighted median | 26 | -0.073644493 | 0.051752809 | 0.154734781 | -0.175079998 | 0.027791013 | 0.929001902 | 0.839389868 | 1.028180786 |
| | | | | | Inverse variance weighted | 26 | -0.076539802 | 0.034822496 | 0.027949198 | -0.144791894 | -0.008287709 | 0.926316045 | 0.865202328 | 0.991746539 |
| | | | | | Simple mode | 26 | -0.110387125 | 0.08224505 | 0.191602606 | -0.271587424 | 0.050813174 | 0.895487403 | 0.762168649 | 1.05212631 |
| | | | | | Weighted mode | 26 | -0.068195531 | 0.058377486 | 0.253753332 | -0.182615404 | 0.046224342 | 0.934077814 | 0.833088497 | 1.04730934 |
| ebi-a-GCST90001875 | MFI | Maturation stages of T cell | HVEM1000_150630.trait2 | HVEM on CD4+ | MR Egger | 21 | -0.06719112 | 0.058819218 | 0.267507428 | -0.182476787 | 0.048094547 | 0.935016484 | 0.833203985 | 1.049269856 |
| | | | | | Weighted media | 21 | -0.093754964 | 0.049383229 | 0.057627842 | -0.190546094 | 0.003036166 | 0.910505842 | 0.82650766 | 1.00304078 |

| | | | | | | | | | | | | | | |
|---|---|---|---|---|---|---|---|---|---|---|---|---|---|---|
| | | | | | n | | | | | | | | | |
| | | | | | Inverse variance weighted | 21 | -0.074770207 | 0.03628775 | 0.039352612 | -0.145894196 | -0.003646217 | 0.9279567 | 0.864249139 | 0.996360422 |
| | | | | | Simple mode | 21 | -0.118016104 | 0.085269282 | 0.181597763 | -0.285143896 | 0.049111688 | 0.888681741 | 0.75190605 | 1.050337654 |
| | | | | | Weighted mode | 21 | -0.124100202 | 0.065368806 | 0.072160903 | -0.252223062 | 0.004022659 | 0.883291329 | 0.777071384 | 1.00403076 |
| ebi-a-GCST90001892 | MFI | Treg | Blue.585.42.Treg.trait19 | CD28 on CD39+ CD4+ | MR Egger | 19 | -0.089926805 | 0.061078923 | 0.159210484 | -0.209641495 | 0.029787884 | 0.913998083 | 0.810874897 | 1.030235981 |
| | | | | | Weighted median | 19 | -0.058732161 | 0.053459751 | 0.271932137 | -0.163513272 | 0.04604895 | 0.942959297 | 0.849155229 | 1.047125667 |
| | | | | | Inverse variance weighted | 19 | -0.075361732 | 0.038427267 | 0.04986123 | -0.150679175 | -4.43E-05 | 0.927407952 | 0.860123604 | 0.999955712 |

| | | | | | | | | | | | | | | |
|---|---|---|---|---|---|---|---|---|---|---|---|---|---|---|
| | | | | | Simple mode | 19 | 0.01947058 | 0.075596919 | 0.799669589 | -0.128699381 | 0.16764054 | 1.019661368 | 0.879238242 | 1.182511469 |
| | | | | | Weighted mode | 19 | -0.077487659 | 0.052929369 | 0.160444744 | -0.181229222 | 0.026253904 | 0.925438445 | 0.83424411 | 1.026601573 |
| ebi-a-GCST90001923 | MFI | Treg | Blue.670.LP.Treg.trait10 | CD127 on CD45RA- CD4 not Treg | MR Egger | 18 | -0.097596522 | 0.060477779 | 0.126125924 | -0.216132968 | 0.020939924 | 0.907014791 | 0.805628172 | 1.021160703 |
| | | | | | Weighted median | 18 | -0.096184529 | 0.064977576 | 0.138800497 | -0.223540578 | 0.03117152 | 0.908296393 | 0.799682441 | 1.031662439 |
| | | | | | Inverse variance weighted | 18 | -0.108798436 | 0.046246589 | 0.018644073 | -0.199441749 | -0.018155122 | 0.896911185 | 0.819187938 | 0.982008689 |
| | | | | | Simple mode | 18 | -0.103179459 | 0.099601665 | 0.314750847 | -0.298398723 | 0.092039805 | 0.901965093 | 0.742005426 | 1.096408463 |
| | | | | | Weighted mode | 18 | -0.095793665 | 0.05662161 | 0.108927212 | -0.20677202 | 0.015184691 | 0.908651484 | 0.813205023 | 1.015300564 |

| | | | | | | | | | | | | | |
|---|---|---|---|---|---|---|---|---|---|---|---|---|---|
| ebi-a-GCST90001977 | Morphological parameter | TBNK | FSC.TBNK.trait8 | FSC-A on HLA DR+ CD4+ | MR Egger | 17 | -0.177949025 | 0.074589231 | 0.030668436 | -0.324143918 | -0.031754132 | 0.836985088 | 0.723146161 | 0.968744737 |
| | | | | | Weighted median | 17 | -0.132445123 | 0.076914159 | 0.085072013 | -0.283196875 | 0.018306629 | 0.875951002 | 0.753371454 | 1.018475222 |
| | | | | | Inverse variance weighted | 17 | -0.125212977 | 0.055151328 | 0.02318618 | -0.23330958 | -0.017116374 | 0.882308971 | 0.791908376 | 0.983029278 |
| | | | | | Simple mode | 17 | -0.120229556 | 0.1293497 | 0.366446066 | -0.373754969 | 0.133295856 | 0.886716862 | 0.688145508 | 1.14258799 |
| | | | | | Weighted mode | 17 | -0.176202179 | 0.080665842 | 0.044165433 | -0.334307228 | -0.018097129 | 0.83844845 | 0.715833824 | 0.982065641 |
| ebi-a-GCST90002011 | MFI | Monocyte | mono.trait6 | CD64 on CD14+ CD16+ monocyte | MR Egger | 13 | 0.009607455 | 0.178592831 | 0.958062834 | -0.340434494 | 0.359649404 | 1.009653755 | 0.71146113 | 1.432826983 |
| | | | | | Weighted | 13 | -0.10772 | 0.120202116 | 0.370159474 | -0.34331 | 0.127874054 | 0.897877088 | 0.709412412 | 1.136409867 |

| | | | | | | | | | | | | | |
|---|---|---|---|---|---|---|---|---|---|---|---|---|---|
| | | | | | median | | 2093 | | | 824 | | | | |
| | | | | | Inverse variance weighted | 13 | -0.188929959 | 0.088037973 | 0.031872553 | -0.361484385 | -0.016375533 | 0.827844488 | 0.696641474 | 0.983757817 |
| | | | | | Simple mode | 13 | -0.066886705 | 0.158304526 | 0.680118174 | -0.377163576 | 0.243390167 | 0.93530116 | 0.685803883 | 1.27556621 |
| | | | | | Weighted mode | 13 | -0.066886705 | 0.133987024 | 0.626662386 | -0.329501272 | 0.195727862 | 0.93530116 | 0.719282371 | 1.216195887 |
| ebi-a-GCST90002037 | MFI | cDC | Red.780.60.cDC.trait3 | CD80 on plasmacytoid DC | MR Egger | 21 | 0.084738942 | 0.053514829 | 0.129818728 | -0.020150122 | 0.189628007 | 1.088432886 | 0.980051535 | 1.208799849 |
| | | | | | Weighted median | 21 | 0.10045531 | 0.054588458 | 0.065734499 | -0.006538068 | 0.207448689 | 1.105674229 | 0.993483259 | 1.230534575 |
| | | | | | Inverse variance weigh | 21 | 0.12072165 | 0.038536495 | 0.001732313 | 0.045190119 | 0.196253181 | 1.128310803 | 1.046226748 | 1.216834946 |

| | | | | | | | | | | | | | | |
|---|---|---|---|---|---|---|---|---|---|---|---|---|---|---|
| | | | | | ted | | | | | | | | | |
| | | | | | Simple mode | 21 | 0.128867162 | 0.076856393 | 0.109157911 | -0.021771368 | 0.279505693 | 1.137539006 | 0.978463917 | 1.322475941 |
| | | | | | Weighted mode | 21 | 0.109306856 | 0.051513492 | 0.046528911 | 0.008340411 | 0.210273301 | 1.115504597 | 1.008375289 | 1.234015271 |
| ebi-a-GCST90002038 | MFI | cDC | Red.780.60.cDC.trait4 | CD80 on CD62L+ plasmacytoid DC | MR Egger | 21 | 0.053778936 | 0.055590731 | 0.345493795 | -0.055178897 | 0.162736769 | 1.055251299 | 0.94631584 | 1.176726898 |
| | | | | | Weighted median | 21 | 0.102656056 | 0.055390401 | 0.063836721 | -0.005909131 | 0.211221243 | 1.108110215 | 0.994108294 | 1.235185601 |
| | | | | | Inverse variance weighted | 21 | 0.103576151 | 0.03942255 | 0.008605717 | 0.026307953 | 0.180844349 | 1.109130251 | 1.026657062 | 1.19822866 |
| | | | | | Simple mode | 21 | 0.120486385 | 0.089904383 | 0.195220491 | -0.055726207 | 0.296698976 | 1.128045382 | 0.945798054 | 1.345410238 |
| | | | | | Weighted | 21 | 0.08755846 | 0.054625631 | 0.124637171 | -0.01950 | 0.194624696 | 1.091506072 | 0.980681268 | 1.214854962 |

| | | | | | | | | | | | | | | |
|---|---|---|---|---|---|---|---|---|---|---|---|---|---|---|
| | | | | | mode | | | | | 7777 | | | | |
| ebi-a-GCST90002058 | MFI | TBNK | Red.780.60.TBNK.trait1 | CD8 on CD8br | MR Egger | 30 | -0.138213608 | 0.067199785 | 0.049134931 | -0.269925187 | -0.006502028 | 0.870912638 | 0.763436607 | 0.993519064 |
| | | | | | Weighted median | 30 | -0.123066093 | 0.06382952 | 0.053849712 | -0.248171953 | 0.002039766 | 0.884205221 | 0.78022577 | 1.002041848 |
| | | | | | Inverse variance weighted | 30 | -0.120217769 | 0.042497088 | 0.004671599 | -0.203512061 | -0.036923478 | 0.886727314 | 0.815860364 | 0.963749881 |
| | | | | | Simple mode | 30 | -0.125682688 | 0.102638622 | 0.230611881 | -0.326854388 | 0.075489011 | 0.881894638 | 0.721188749 | 1.078411377 |
| | | | | | Weighted mode | 30 | -0.143365817 | 0.075081602 | 0.066139843 | -0.290525756 | 0.003794122 | 0.866437053 | 0.747870267 | 1.003801329 |
| ebi-a-GCST90002068 | MFI | Treg | Red.780.60.Treg.trait7 | CD4 on secreting Treg | MR Egger | 28 | -0.104257185 | 0.060832701 | 0.098459069 | -0.22348928 | 0.014974909 | 0.900993545 | 0.799723465 | 1.015087595 |
| | | | | | Weighted media | 28 | -0.111972518 | 0.055311438 | 0.04292893 | -0.220382936 | -0.003562101 | 0.894068828 | 0.802211544 | 0.996444236 |

| | | | | | | | | | | | | | | |
|---|---|---|---|---|---|---|---|---|---|---|---|---|---|---|
| | | | | | n | | | | | | | | | |
| | | | | | Inverse variance weighted | 28 | -0.086557134 | 0.039234947 | 0.027375363 | -0.163457631 | -0.009656637 | 0.91708315 | 0.849202478 | 0.990389838 |
| | | | | | Simple mode | 28 | -0.048643061 | 0.103736595 | 0.642898611 | -0.251966787 | 0.154680666 | 0.952521061 | 0.777270553 | 1.167285148 |
| | | | | | Weighted mode | 28 | -0.112388611 | 0.068635393 | 0.113135624 | -0.246913982 | 0.02213676 | 0.893696889 | 0.781207888 | 1.022383596 |
| ebi-a-GCST90002082 | Morphological parameter | TBNK | SSC.TBNK.trait5 | SSC-A on CD8br | MR Egger | 19 | 0.170894682 | 0.064985919 | 0.017564895 | 0.043522281 | 0.298267082 | 1.186365797 | 1.044483267 | 1.347521639 |
| | | | | | Weighted median | 19 | 0.155453407 | 0.073988531 | 0.03563681 | 0.010435887 | 0.300470927 | 1.168187505 | 1.01049053 | 1.350494642 |
| | | | | | Inverse variance weigh | 19 | 0.150288784 | 0.051436625 | 0.003479877 | 0.049472999 | 0.251104569 | 1.16216981 | 1.050717221 | 1.285444495 |

| | | | | | | | | | | | | | | |
|---|---|---|---|---|---|---|---|---|---|---|---|---|---|---|
| | | | | | ted | | | | | | | | | |
| | | | | | Simpl e mode | 19 | - 0.07664 8434 | 0.13438 2256 | 0.57547 4093 | - 0.34003 7656 | 0.18674 0788 | 0.92621 5422 | 0.71174 3521 | 1.20531 4812 |
| | | | | | Weig hted mode | 19 | 0.16308 9555 | 0.07874 3977 | 0.05299 6575 | 0.00875 1361 | 0.31742 7749 | 1.17714 2104 | 1.00878 9766 | 1.37358 9998 |

| Table S3. Analysis for causal associations of thyroid cancer risk on immune cells by IVW method | | | | | | | | | | | | | | |
|---|---|---|---|---|---|---|---|---|---|---|---|---|---|---|
| Data source | Trait type | Panel | Statistical trait name | Trait | method | nsnp | b | se | pval | lo_ci | up_ci | or | or_lci95 | or_uci95 |
| ebi-a-GCST90001639 | Absolute count | TBNK | TB_Trait560 | CD3-lymphocyte AC | Inverse variance weighted | 14 | 0.062550752 | 0.025721678 | 0.015022751 | 0.012136263 | 0.112965241 | 1.064548485 | 1.012210206 | 1.119593016 |
| ebi-a-GCST90001645 | Absolute count | TBNK | TB_Trait566 | NK AC | Inverse variance weighted | 14 | 0.050719371 | 0.024993576 | 0.04242812 | 0.001731962 | 0.09970678 | 1.052027623 | 1.001733463 | 1.104846907 |
| ebi-a-GCST90001789 | MFI | B cell | Bcells.trait504 | CD25 on IgD- CD38dim | Inverse variance weighted | 14 | -0.050898589 | 0.025162051 | 0.043090525 | -0.10021621 | -0.001580969 | 0.950375044 | 0.904641804 | 0.99842028 |

**Table S4. Analysis for causal associations of thyroid cancer risk on immune cells by five MR methods**

| Data source | Trait type | Panel | Statistical trait name | Trait | method | nsnp | b | se | pval | lo_ci | up_ci | or | or_lci95 | or_uci95 |
|---|---|---|---|---|---|---|---|---|---|---|---|---|---|---|
| ebi-a-GCST90001639 | Absolute count | TBNK | TB_Trait 560 | CD3-lymphocyte AC | MR Egger | 14 | 0.009675347 | 0.053712685 | 0.860055439 | -0.095601514 | 0.114952209 | 1.009722305 | 0.908826098 | 1.121819824 |
| | | | | | Weighted median | 14 | 0.060581322 | 0.035182703 | 0.085086469 | -0.008376775 | 0.129539419 | 1.062453995 | 0.991658212 | 1.138303981 |
| | | | | | Inverse variance weighted | 14 | 0.062550752 | 0.025721678 | 0.015022751 | 0.012136263 | 0.112965241 | 1.064548485 | 1.012210206 | 1.119593016 |
| | | | | | Simple mode | 14 | 0.079113678 | 0.042131588 | 0.083026835 | -0.003464234 | 0.16169159 | 1.082327352 | 0.99654176 | 1.175497651 |
| | | | | | Weighted mode | 14 | 0.064680027 | 0.036926863 | 0.103388504 | -0.007696625 | 0.137056679 | 1.066817617 | 0.992332918 | 1.146893152 |
| ebi-a-GCST90001645 | Absolute count | TBNK | TB_Trait 566 | NK AC | MR Egger | 14 | 0.018664308 | 0.052240741 | 0.727090404 | -0.083727545 | 0.121056161 | 1.018839575 | 0.919681794 | 1.128688298 |
| | | | | | Weighted median | 14 | 0.025060385 | 0.033036766 | 0.448115535 | -0.039691678 | 0.089812447 | 1.025377036 | 0.961085718 | 1.093969087 |
| | | | | | Inverse variance | 14 | 0.050719371 | 0.024993576 | 0.04242812 | 0.001731962 | 0.09970678 | 1.052027623 | 1.001733463 | 1.104846907 |

| | | | | | | | | | | | | | | |
|---|---|---|---|---|---|---|---|---|---|---|---|---|---|---|
| | | | | | weighted | | | | | | | | | |
| | | | | | Simple mode | 14 | 0.049545015 | 0.046024515 | 0.301279623 | -0.040663034 | 0.139753065 | 1.050792893 | 0.960152614 | 1.149989791 |
| | | | | | Weighted mode | 14 | 0.019789822 | 0.037989686 | 0.611176963 | -0.054669962 | 0.094249606 | 1.019986939 | 0.946797576 | 1.098833987 |
| ebi-a-GCST90001789 | MFI | B cell | Bcells.trait504 | CD25 on IgD- CD38dim | MR Egger | 14 | -0.091336523 | 0.052504291 | 0.107488706 | -0.194244934 | 0.011571888 | 0.912710511 | 0.823456187 | 1.011639101 |
| | | | | | Weighted median | 14 | -0.04997306 | 0.035303071 | 0.156908979 | -0.119167079 | 0.019220959 | 0.951255051 | 0.887659479 | 1.019406871 |
| | | | | | Inverse variance weighted | 14 | -0.050898589 | 0.025162051 | 0.043090525 | -0.10021621 | -0.001580969 | 0.950375044 | 0.904641804 | 0.99842028 |
| | | | | | Simple mode | 14 | -0.089043615 | 0.048393333 | 0.088706517 | -0.183894548 | 0.005807318 | 0.914805674 | 0.832023538 | 1.005824213 |
| | | | | | Weighted mode | 14 | -0.045300567 | 0.039163384 | 0.268205908 | -0.122060799 | 0.031459664 | 0.955710183 | 0.885094554 | 1.03195975 |
