# Supplementary material for: Investigating the causal relationship between 731 immune phenotypes and thyroid cancer risk: A bidirectional Mendelian randomization study
Source: Medicine (Baltimore). 2025 Oct 17;104(42):e45072. doi: 10.1097/MD.0000000000045072 (PMC12537243; doi:10.1097/MD.0000000000045072)

Figure S1 Scatter plots and funnel plots of the above results indicate stability.

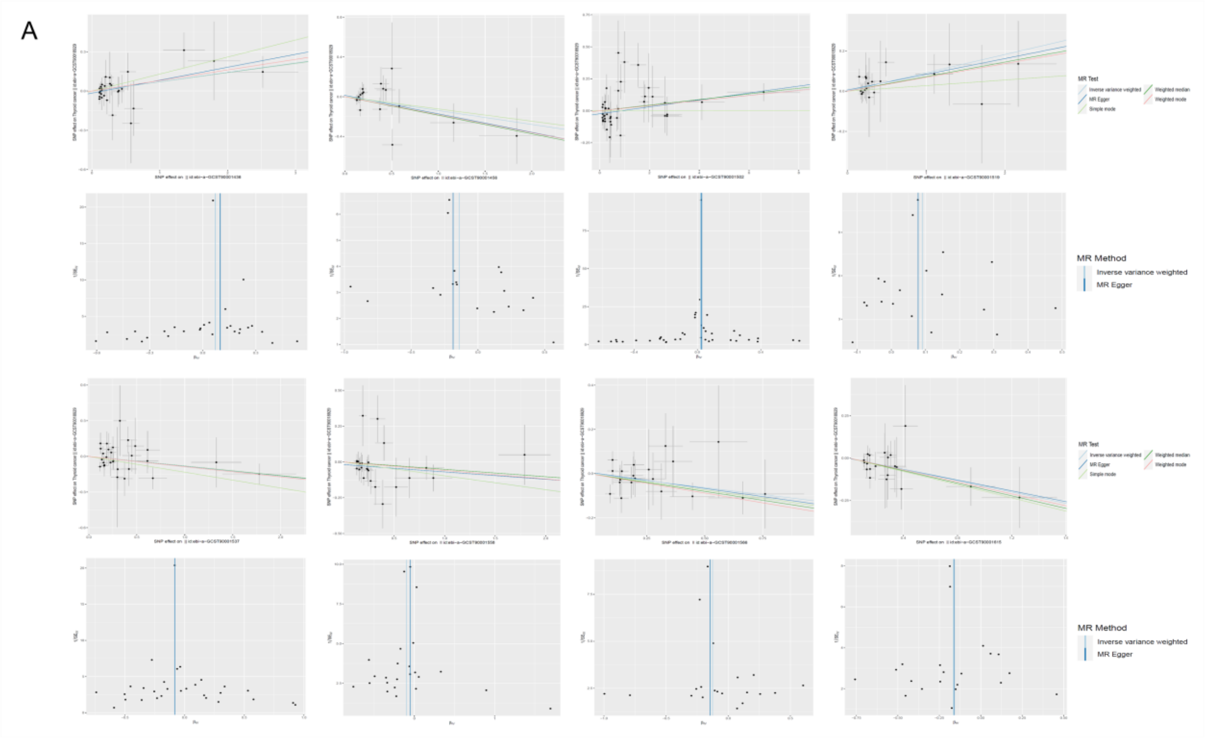

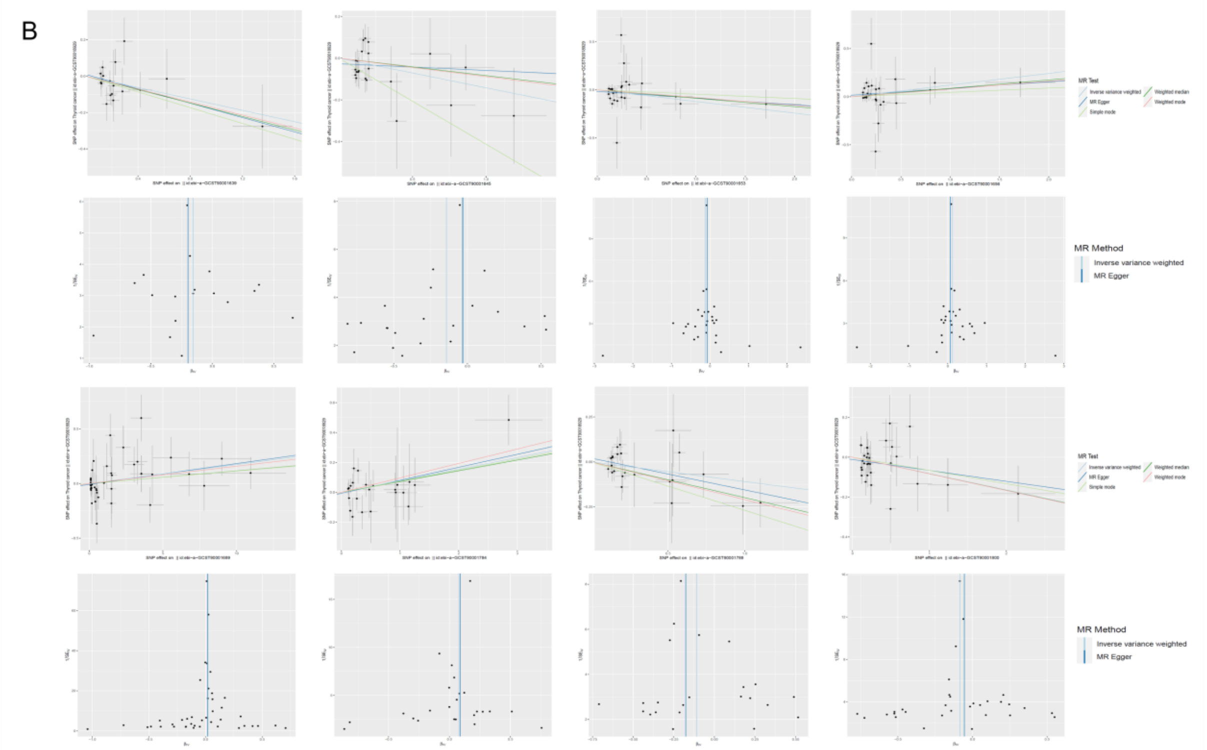

C

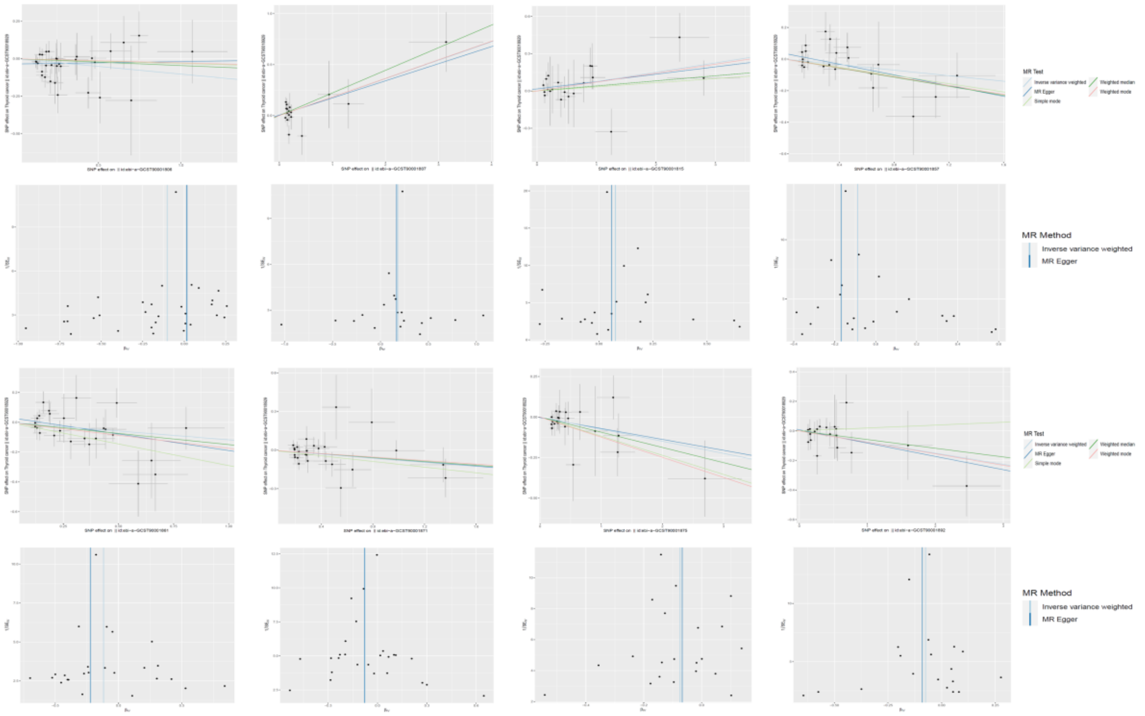
MR Test
Inverse variance weighted
MR Egger
Simple mode
Weighted median
Weighted mode
MR Method
Inverse variance weighted
MR Egger

D

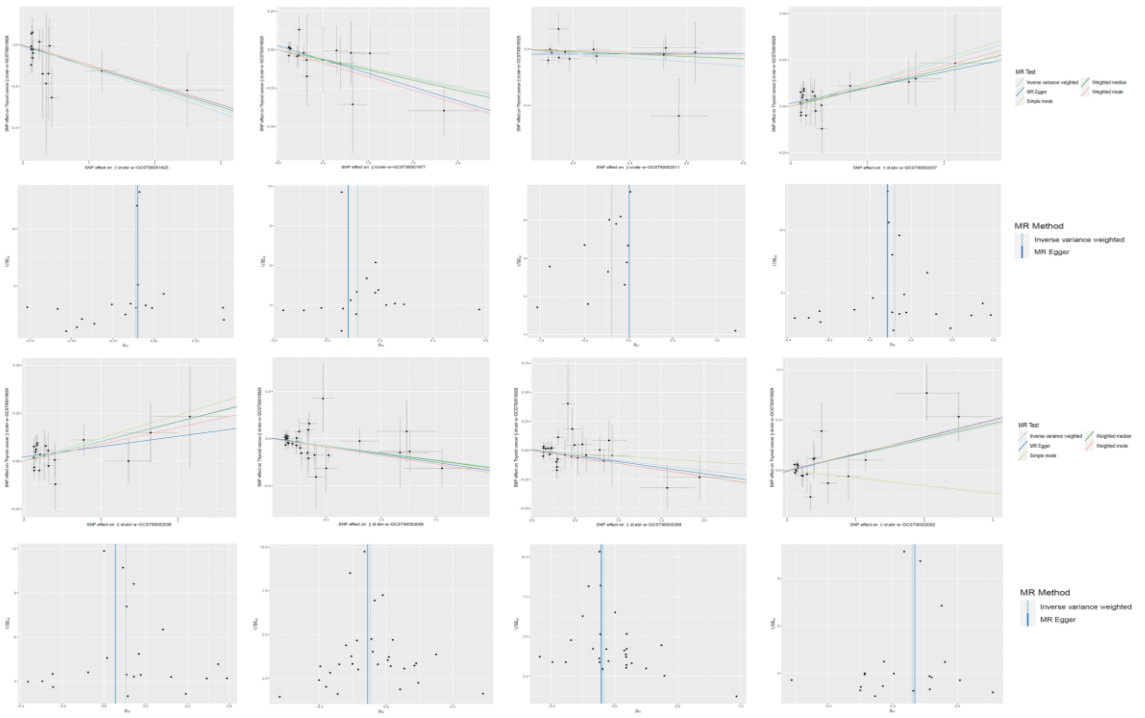
MR Test
Inverse variance weighted
MR Egger
Simple mode
Weighted median
Weighted mode
MR Method
Inverse variance weighted
MR Egger

Figure S2 The “leave-one-out” method of the above results indicates stability.

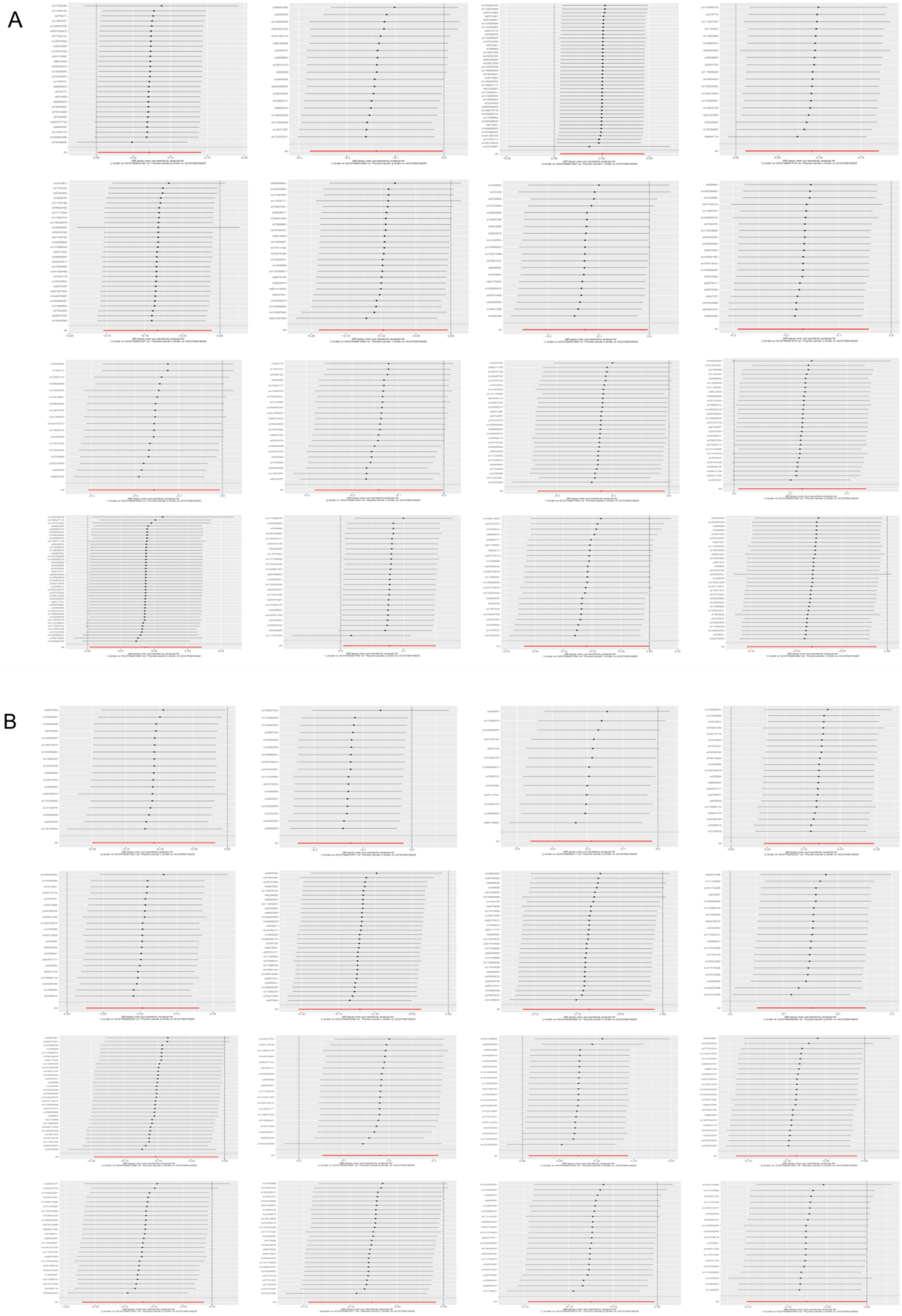

Figure S3 Scatter plots and funnel plots of the above results indicate stability.

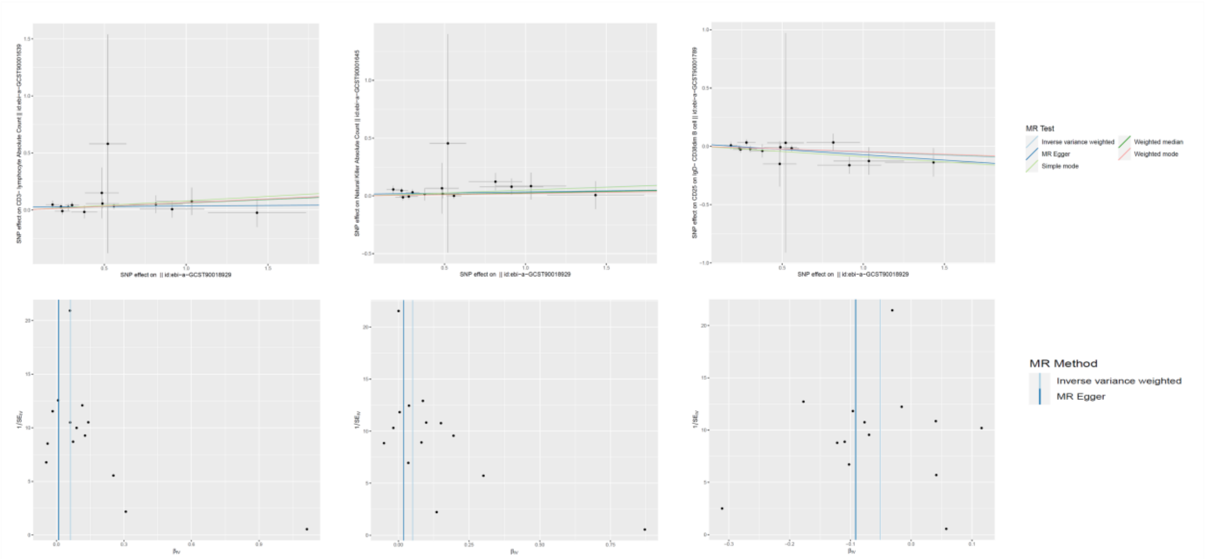

Figure S4 The “leave-one-out” method of the above results indicates stability.

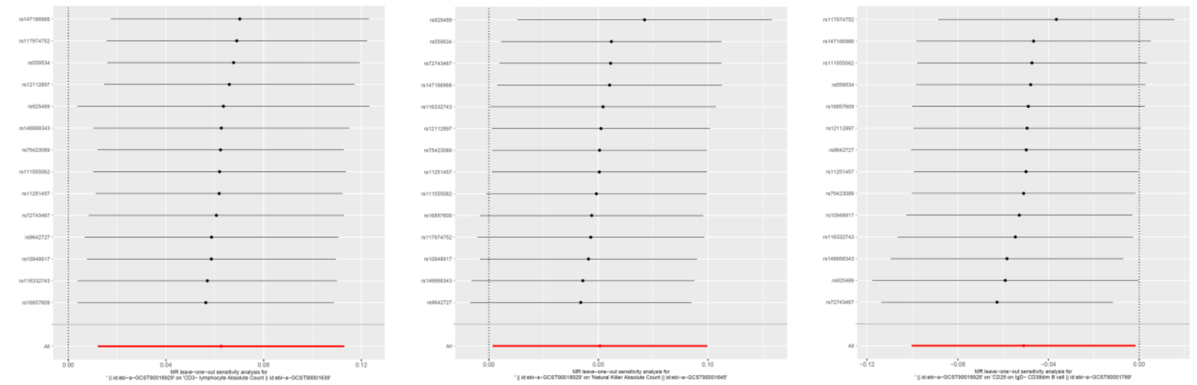

Supplement: Supplementary file 2 [file medi-104-e45072-s002.pdf]
